# FMT from Exercise and Konjac Glucomannan Preconditioned Donors Rescues Antibiotic-Induced Dysbiosis with Enhanced Ecological Restoration in Mice

**DOI:** 10.3390/nu18101544

**Published:** 2026-05-13

**Authors:** Minghan Wang, Yanan Lyu, Jiangyu Zhang, Yanyan Wang, Yan Yang, Yu-Heng Mao

**Affiliations:** 1 School of Exercise and Health, Guangzhou Sport University, Guangzhou 510500, China; 2 School of Public Health (Shenzhen), Shenzhen Campus of Sun Yat-sen University, Shenzhen 518107, China; 3 Guangdong Engineering Technology Research Center of Nutrition Transformation, Sun Yat-sen University, Shenzhen 518107, China; 4 Guangdong Provincial Key Laboratory of Food, Nutrition and Health, Sun Yat-sen University, Guangzhou 510080, China; 5 Guangzhou Center for Disease Control and Prevention (Guangzhou Health Supervision Institute), Guangzhou 510440, China; 6 Guangdong Key Laboratory of Human Sports Performance Science, Guangzhou Sport University, Guangzhou 510500, China

**Keywords:** FMT, exercise, KGM, dysbiosis, arginine metabolism

## Abstract

Background: Although antibiotics have a wide range of applications in medical clinical practice and possess significant clinical value, their inevitable contribution to gut microbiome dysbiosis warrants attention. Our previous research has confirmed that the combined intervention of exercise and konjac glucomannan (KGM) has a better regulatory effect on gut dysbiosis in mice compared with individual interventions. Methods: This study aims to further investigate whether this effect can be transmitted through fecal microbiota transplantation (FMT), and to compare the recovery effects of autologous FMT (a-FMT), fecal microbiota transplantation after exercise combined with KGM intervention (EK-FMT), and combinative intervention with exercise and KGM (EXE-KGM) on gut microbiome dysbiosis. Sample sizes ranged from five to six animals. Results: The results showed that the a-FMT group recovered α diversity the fastest, including Chao, Shannon, and Simpson indices(*p* < 0.05), within 2 weeks after transplantation when compared with the CTL group. At the end of the experiment, the Bray–Curtis distance of the a-FMT group was closest to the CTL group, while the EXE-KGM group had delayed recovery, there was no significant difference between the EK-FMT group and the EXE-KGM group. Metagenomic analysis and metabolomics analysis indicated that the arginine synthesis and metabolism pathways (KEGG: map00471, map00473, arginine biosynthesis) played a core role in the restoration of the microbiota. Conclusions: The results of this experiment indicate that EK-FMT group can partially transfer the regulatory effects of combined exercise and KGM intervention, a-FMT accelerates the recovery speed of the gut microbiome and arginine metabolism may play an important role in it. This finding provides a theoretical basis and practical direction for special populations to receive special donor fecal treatment.

## 1. Introduction

Antibiotics provide effective treatment for bacterial infections, significantly reducing the incidence and mortality rates associated with infectious diseases [1]. However, the widespread use of antibiotics has brought many adverse consequences, among which damage to the gut microbiome caused by antibiotics is particularly prominent [2]. The gut microbiome is a complex ecosystem composed of trillions of microorganisms that exist in the gastrointestinal tract and plays a crucial role in human health, participating in digestion, nutrient absorption, immune system regulation, etc. [3]. Gut microbiome dysbiosis can lead to a series of health problems, including increased susceptibility to infections, metabolic disorders, and inflammatory diseases, such as inflammatory bowel disease [4].

Currently, the main approaches to address the gut microbiome dysbiosis caused by antibiotics are through dietary modifications, the use of microecologics, and even natural recovery methods [5,6]. Microecologics include probiotics, prebiotics, and synbiotics, etc. They mainly affect the composition and function of the gut microbiome, improving the balance of the intestinal microecology, thereby regulating the host’s immune response, metabolic state, and intestinal barrier function, and exerting potential beneficial effects in disease prevention and adjunctive treatment [7]. Among them, our studies demonstrated that konjac glucomannan (KGM), as a water-soluble polysaccharide extracted from the tuber of *Amorphophallus konjac C. Koch*, belongs to a type of prebiotic. KGM of varying molecular weights and viscosities differentially modulates the gut microbiome. Specifically, previous studies have demonstrated that high-molecular-weight KGM can effectively regulate gut microbiome dysbiosis, while low-to-medium doses of KGM can effectively alleviate appetite suppression induced by excessive exercise [8,9,10,11,12]. Besides these microecologics, lifestyle adjustments, such as exercise, have also been extensively studied to positively regulate the gut microbiome and, finally, on metabolic function [12,13,14,15,16]. Our previous research demonstrated that exercise, combined with KGM, can effectively alleviate gut microbiome dysbiosis in mice receiving antibiotic treatment [17].

However, individuals who are non-ambulatory or have restricted activity (e.g., due to fractures, critical illness, or cardiopulmonary diseases) are often at risk of dysbiosis of the gut microbiome. In such cases, particularly when necessary medical treatments like antibiotics are involved, autologous fecal microbiota transplantation (FMT) may represent an effective and appropriate approach for regulating gut microbiome dysbiosis [18]. FMT refers to introducing the fecal matter of a healthy donor into the recipient’s gut to introduce a new gut microbiome and restore the balance of the gut microbiome. In particular, it is clinically used to treat recurrent *Clostridioides difficile* infection (CDI), with a cure rate approaching 90% [19]. In addition, FMT is also being explored for other diseases such as obesity and neurological disorders [20,21,22], highlighting its potential as a multifunctional therapeutic tool. Autologous FMT (a-FMT), as a special type of FMT, which involves banking one’s feces during a healthy state for later use in restoring the gut microbiome following perturbation, has shown outstanding effects in various diseases [23]. For example, it has significant improvement effects for patients with ulcerative colitis, obese individuals, and children with Crohn’s disease [24,25,26]. Compared with heterologous FMT, autologous FMT has a relatively strong regulatory effect on intestinal diseases and avoids the colonization resistance of allogeneic FMT, reducing the risk of infection, which is particularly important for high-risk groups such as the elderly, patients with comorbidities, and patients with low immune function. However, the storage of donor feces is not a common practice. It should be noted that long-term storage of feces may compromise bacterial viability [27]. Consequently, a-FMT often lacks the essential prerequisite of pre-stored feces collected during a healthy state, which significantly restricts its feasibility and broader application in most clinical or experimental contexts.

Based on our previous study on the effectiveness of KGM and exercise combination [17], this study hypothesizes that the effects can be retained and transmitted through FMT. The aim of this study is to analyze the differences among various strategies for regulating gut microbiome dysbiosis after antibiotic treatment. A-FMT methods were utilized for comparative analysis. The findings establish a foundational rationale for the development of non-invasive, microbiota-targeted strategies, underscoring a novel approach for translating lifestyle interventions into practice.

## 2. Materials and Methods

### 2.1. Chemicals

In accordance with our prior work [17], natural KGM with the highest molecular weight was selected for the present investigation. KGM with a purity of over 95% was purchased from Johnson & Johnson (Ezhou, China). The KGM employed in this study possessed a molecular weight of 1.82 × 10^7^ Da, a specific viscosity of 9.48 dL/g, and a galactose-to-glucose ratio of 1.65:1. Six standard short-chain fatty acids (SCFAs), namely acetic acid, propionic acid, *n*-butyric acid, *iso*-butyric acid, *n*-valeric acid, and *iso*-valeric acid, were obtained from Aladdin (Shanghai, China). A DNA extraction kit was procured from Tiangen Biotechnology Co., Ltd. (Beijing, China). All chemicals utilized in this study are listed in Table S1, with their detailed information sourced from the NCBI PubChem Compound Database and the respective suppliers.

### 2.2. Animal Design

**Figure 1:**
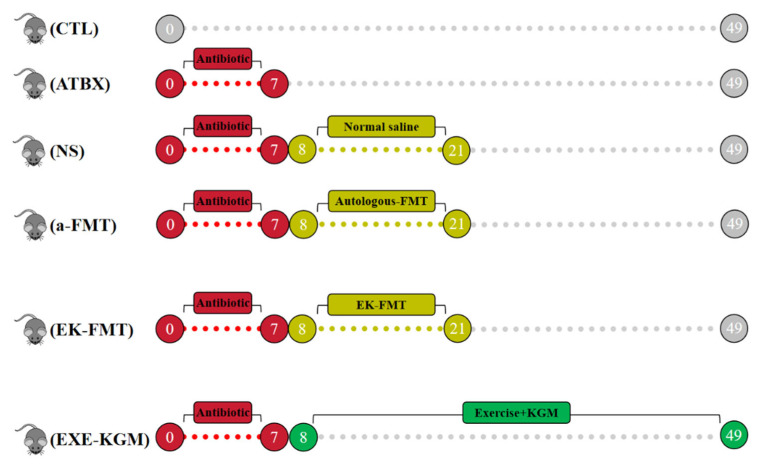
Flow chart of animal experiment. Different numbers indicate different days after antibiotic intervention.

Figure 1 shows the flowchart of this experiment. Before the formal experiment began, the feces of the a-FMT group of mice were collected, and eight healthy C57BL/6J mice were selected for the exercise combined with KGM intervention (EK-Donor group) experiment. Their feces were also collected for the formal FMT experiment.

In the formal experiment, thirty-six 10-week-old male C57BL/6J mice were purchased from Zhuhai BesTest Bio-Tech Co., Ltd. (Zhuhai, China) and randomly divided into control group (CTL), antibiotic group (ATBX), gavage saline group (NS) (in the NS group, a mouse experienced a rapid 20% weight loss due to scratch, therefore was euthanized during the experiment. Statistical analyses demonstrated that the absence of one animal did not compromise the validity of the experimental findings. The η^2^ values were all >0.14 at each time point [28]), autology-FMT group (a-FMT), exercise combined with KGM intervention mice FMT group (EK-FMT), and exercise combined with KGM intervention (EXE-KGM). The mice were preconditioned one week before the formal experiment.

Except for the EXE-KGM and the EK-Donor groups, the other five groups of mice were fed freely with ultra-pure water. EXE-KGM group and EK-Donor group mice were added to KGM with a concentration of 2.5 g/L in drinking water. In the first week, except for the CTL group, the other 5 groups of mice were administered antibiotics in drinking water or KGM mixed solution, antibiotic formulation, and reference previous studies (Ampicillin, streptomycin, and clindamycin at a ratio of 1 mg/mL each) [17]. During weeks 2–3, the mice in the a-FMT group underwent autologous FMT, and the mice in the EK-FMT group underwent EK-Donor, after thawing the donor feces, adding 9% normal saline to it in a 1:3 ratio, and vortex mixing. Then centrifuge at 2000× *g* for 5 min and take the supernatant. Administer this solution by gavage to the mice once a day. The mice in the NS group were intragastrically administered 9% normal saline at the same frequency. Throughout the experiment, the mice drank and ate freely. Drinking water was refreshed every 2–3 days, and water intake was recorded every 2–3 days; food intake was calculated once a week. The average daily food and average daily water intakes were calculated for each mouse throughout the experiment. The mice exercised on a ZH-PT/5S treadmill, and the EK and EXE-KGM groups underwent a 6-week training regimen. As shown in Table 1, the mice in the EK-Donor and EXE-KGM groups (after antibiotic intervention) underwent moderate-intensity exercise intervention. Before and after each exercise session, 5 min of warm-up and 5 min of relaxation are carried out. The actual exercise duration is 30 min. To ensure that the mice maintained a moderate exercise intensity (60% of their maximum running speed), maximum running speed tests were conducted using a documented method [17] before the exercise intervention and three weeks after the exercise intervention.

Feces samples from all groups were collected in sterile centrifuge tubes at day 0, 7, 21, 35, and 49 and stored in a −80 °C refrigerator for subsequent analysis. After an endurance test at the experimental endpoint, the mice were given a day’s rest and subsequently anesthetized with sodium pentobarbital. A needle is used to take a blood sample from the heart. The sample was centrifuged at 4 °C at 3000 rpm for 15 min, and then the serum was collected and stored in a refrigerator at −80 °C for subsequent analysis.

The 3R principle was strictly adhered to throughout all experimental procedures. If the mice exhibited poor physical condition, stool sampling and exercise interventions were immediately halted until their bodily status recovered to normal. To exclude the potential confounding impact of estrogen on experimental results, only male mice were selected for the present study. The mice were housed in a specific pathogen-free (SPF) facility at the Animal Center of Guangzhou Sport University, under standard rearing conditions (temperature: 22–24 °C, relative humidity: 50 ± 5%) with a 12 h light/dark cycle implemented consistently. All experimental operations were conducted in compliance with the NRC Guidelines for the Care and Use of Laboratory Animals (2011). During the entire study period, the mice were supplied with standard laboratory feed (AIN-93G purified diet; the detailed formulation of which is presented in Table S2) and distilled water ad libitum. This animal experiment was reviewed and approved by the Animal Experimental Ethics Review Committee of Guangzhou Sport University, with the official approval number: 2024DWLL-47.

### 2.3. Endurance Test

After running at a speed of 10 m/min for 10 min, the running speed increased by 2 m/min every 2 min until the mice reached exhaustion. The treadmill speed and distance at this time were recorded. The definition of the point of exhaustion is that a mouse is exposed to the electric grid at least five times within one minute or that a mouse is unable to persist on a treadmill for 10 s under electrical stimulation [29,30].

### 2.4. Procedures for FMT

The FMT protocol was referred to the previous literature with a small modification [27]. Fecal sample collection was performed from 7:00 to 11:00 in the early morning, mainly to minimize the effects of circadian rhythm on the fluctuation of gut microbiome abundance [31]. Additionally, the area for fecal sample collection is disinfected before sampling. Mice were raised normally according to the standard feeding protocol without any special treatment. After gentle fixation, fresh fecal pellets were collected immediately upon excretion and allowed to drop directly into sterile centrifuge tubes to avoid contamination with urine, bedding, hair, or other impurities. The feces collected in the early stage were collected in sterile centrifuge tubes and stored in a refrigerator at −80 °C. The entire collection process was conducted in strict accordance with the animal experimental ethical requirements of Guangzhou Sport University, and sampling was stopped immediately if any discomfort or abnormal conditions occurred in the mice. A fecal bacterial suspension was prepared by thoroughly mixing feces at a 1:3 ratio with physiological saline. It was then centrifuged at a speed of 2000× *g* for 5 min in a 4 °C centrifuge to retain the supernatant. Throughout this process, all procedures were conducted under anaerobic conditions. The supernatant was then administered by gavage, with 200 μL administered to each mouse; the NS group mice were administered the same volume of physiological saline. The FMT was carried out for two weeks, once a day.

### 2.5. Histological Analysis

Following a rinse with physiological saline, the colon tissues were fixed in a pre-cooled 4% paraformaldehyde solution for a duration of 24 h. Subsequent to fixation, the colon tissues were embedded in paraffin wax and subjected to hematoxylin-eosin (HE) staining. Histological assessments were carried out utilizing the Pannoramic 250 FLASH imaging system (3DHISTECH Ltd., Budapest, Hungary), and intestinal histological scoring was conducted in accordance with the criteria presented in Table S3. Variations in intestinal pathological alterations across the different experimental groups were analyzed with CaseViewer Software Version 2.4 [17].

### 2.6. Analysis of Inflammatory Factors in Colon Tissue

The expressions of inflammation-related factors in colon tissue, including TNF-α, IL-17A, and IL-10, was quantified using commercial kits (Quanzhou Ruixin Biotechnology Co., Ltd., Quanzhou, China) according to the instructions from the manufacturers.

### 2.7. Determination of Short-Chain Fatty Acids (SCFAs)

Concentrations of SCFAs in feces were analyzed by gas chromatography (GC) as previously described [8]. Briefly, feces samples were thoroughly homogenized with distilled water (1:15 *w*/*v*) and then centrifuged at 12,000 rpm for 20 min at 4 °C. The supernatant was collected and acidified to pH 2–3 using 1 M HCl. An Agilent 8890B gas chromatography system equipped with a flame ionization detector (FID) (Agilent Technologies Inc., Santa Clara, CA, USA) was employed for SCFA quantification. Separation was performed using a fused silica capillary column (30 × 0.32 mm, DB-FFAP 123-3232, Agilent) with nitrogen as the carrier gas at a flow rate of 0.6 mL/min. The injection port and detector temperatures were set at 200 °C and 220 °C, respectively, and the injection volume was 1 µL. Six SCFA standards, including acetic acid, propionic acid, *n*-butyric acid, iso-butyric acid, *n*-valeric acid, and *iso*-valeric acid (Aladdin, Shanghai, China), were used for identification and quantification. During the analysis, butyric acid was calculated as the sum of *n*-butyric acid and *iso*-butyric acid, and valeric acid was calculated as the sum of *n*-valeric acid and *iso*-valeric acid.

### 2.8. DNA Extraction and 16S rRNA Sequencing

Genomic DNA was extracted from fecal samples using the Tiangen Fecal DNA Extraction Kit (Tiangen Biotech Co., Ltd., Beijing, China) in strict accordance with the manufacturer’s operational protocols. The concentration of the extracted microbial genomic DNA was quantified with a NanoDrop 2000 spectrophotometer (Thermo Fisher Scientific, Waltham, MA, USA), and 16S rRNA gene sequencing of fecal samples was undertaken by Wekemo Technology Co., Ltd. (Shenzhen, China).

Genomic DNA of the samples was isolated via the CTAB method, and the purity and concentration of the extracted DNA were assessed by 1% agarose gel electrophoresis. An appropriate aliquot of each sample was transferred to centrifuge tubes and diluted to a final concentration of 1 ng/µL using sterile deionized water. The V4 hypervariable region of the 16S rRNA gene was amplified with specific barcoded primers, with the primer sequences as follows: 515 F (GTGCCAGCMGCCGCGGTAA) and 806 R (GGACTACHVGGGTWTCTAAT). The detailed PCR amplification procedure was implemented as follows: (1) Template: diluted genomic DNA of the samples. (2) Primers: Barcoded specific primers were selected according to the target sequencing region, with primer sequences and sequencing regions varying by experimental project and thus determined based on actual research requirements. (3) Enzymatic system and buffers: The Phusion^®^ High-Fidelity PCR Master Mix with GC Buffer (New England Biolabs, Ipswich, MA, USA) was adopted, and high-fidelity DNA polymerase was used for PCR amplification to ensure the efficiency and accuracy of target fragment amplification. (4) PCR reaction system (30 µL total volume): 15 µL of 2× Phusion Master Mix, 1 µL of 1 µM forward primer, 1 µL of 1 µM reverse primer, 10 µL of 1 ng/µL genomic DNA (10 ng total), and 2 µL of nuclease-free water. PCR reaction program: initial denaturation at 98 °C for 1 min, followed by 30 cycles of denaturation at 98 °C for 10 s, annealing at 50 °C for 30 s, and extension at 72 °C for 30 s, with a final extension step at 72 °C for 5 min; (5) PCR thermal cycler: Bio-Rad T100 Gradient PCR Thermal Cycler(Bio-Rad Laboratories, Inc., Hercules, CA, USA).

Based on the quantified concentrations of the PCR amplicons, equal volumes of the PCR products from each sample were pooled and thoroughly mixed. The mixed PCR products were then purified via 2% agarose gel electrophoresis in 1× TAE buffer, and the target PCR bands were excised from the gel and recovered using the Universal DNA Purification and Recovery Kit (Tiangen Biotech Co., Ltd., Beijing, China).

Libraries for high-throughput sequencing were constructed with the NEB Next^®^ Ultra DNA Library Prep Kit for Illumina. Quality inspection and absolute quantification of the constructed libraries were performed via quantitative real-time PCR (q-PCR) on an Agilent 5400 System. Following strict library qualification, high-throughput on-machine sequencing was performed on an Illumina sequencing platform.

Raw sequencing data (fastq format files) were imported into the QIIME2 pipeline and converted to a processable file format using the QIIME2 Import plugin. Quality control, sequence trimming, denoising, read merging, and chimera removal were subsequently performed with the QIIME2 DADA2 plugin, yielding the final feature table [32]. Representative sequences of ASV were taxonomically annotated by sequence alignment against the pre-trained GREENGENES database (Version 13_8, 99% sequence similarity), which was pre-trimmed to the V4 hypervariable region matching the 515F/806R primer set; thus, a taxonomic annotation table of microbial species was generated [33]. Mitochondrial and chloroplast sequence contaminants were then filtered out from the feature table using the QIIME2 feature-table plugin. Differential abundance analysis of bacterial taxa across groups and individual samples was conducted using multiple statistical approaches, including ANCOM, ANOVA, Kruskal–Wallis test, LEfSe(1.0.8), and DESeq2(1.26.0) [34,35,36]. The α and β diversity matrices were further calculated with the QIIME2 Core-Diversity plugin. The α diversity at the ASV level was evaluated to characterize within-sample microbial diversity using four key indices: Observed OTUs, Chao1, Shannon diversity index, and Faith’s phylogenetic diversity (PD) index. The β diversity was used to assess inter-sample variations in microbial community structure, with the Bray–Curtis dissimilarity distance as the primary metric; the results of the beta diversity analysis were visualized using principal coordinate analysis (PCoA) and non-metric multidimensional scaling (NMDS) plots [17,37].

### 2.9. Metagenomic Sequencing

Genomic DNA from fecal samples was obtained using a CTAB-based extraction procedure. The extracted DNA was then examined for integrity, purity, and concentration with an Agilent 2100 Bioanalyzer to ensure that it met the requirements for library construction. For each qualified sample, sequencing libraries were generated using the NEBNext^®^ Ultra™ DNA Library Prep Kit for Illumina (NEB, Ipswich, MA, USA; Catalog#: E7370L). During library preparation, genomic DNA was randomly fragmented by sonication to an average insert of approximately 350 bp, followed by end repair, adenylation of 3’ ends, ligation of Illumina-compatible full-length adapters, and PCR enrichment. The amplified libraries were purified with the AMPure XP purification system (Beverly, MA, USA), and individual index sequences were introduced to distinguish different samples.

After library construction, the quality of each library was checked using an Agilent 5400 system (Agilent, USA). Library concentrations were further determined by q-PCR and adjusted to 1.5 nM. Libraries that passed quality evaluation were mixed in appropriate proportions according to their effective concentrations and the expected sequencing output. Metagenomic sequencing was performed on an Illumina NovaSeq high-throughput sequencing platform using a paired-end 150-bp sequencing mode. The resulting raw data were used to characterize bacterial, fungal, and viral components in fecal samples.

Before downstream bioinformatic analysis, raw reads were processed to remove low-quality and host-derived sequences. Quality control was carried out with Kneaddata, in which Trimmomatic was used for adapter trimming, low-quality read filtering, and length filtering. Adapter contamination was removed using the ILLUMINACLIP parameter with adapters path: 2:30:10. Reads with poor base quality were filtered with the SLIDINGWINDOW:4:20 option, corresponding to a quality threshold of 20, and reads shorter than 50 bp after trimming were discarded using MINLEN:50. To minimize host contamination, the remaining reads were mapped to the host reference genome with Bowtie2 under the very-sensitive mode, and reads aligned to the host genome were excluded. The overall quality of the clean data and the effectiveness of the filtering process were subsequently evaluated with FastQC [38,39,40].

Taxonomic composition was determined using Kraken2 together with a customized microbial reference database. The database was constructed from microbial sequences selected from the NCBI NT nucleotide database and RefSeq whole-genome database, covering bacteria, fungi, archaea, and viruses. Kraken2 assigned taxonomic labels to clean reads using a K-mer classification strategy, while Bracken was used to refine the abundance estimates and calculate the relative abundance of individual taxa. The local Kraken2 database applied in this study contained 16,799 known bacterial genomes [35,41,42,43]. For functional profiling, the host-filtered clean reads were searched against the UniRef90 database using HUMAnN2, with sequence alignment accelerated by the DIAMOND algorithm. Functional annotation results were then converted into abundance profiles for different functional categories according to the correspondence between UniRef90 identifiers and downstream functional databases [36,44,45,46]. Based on the taxonomic and functional abundance matrices, subsequent ecological and multivariate analyses were performed, including abundance clustering, principal coordinate analysis, non-metric multidimensional scaling analysis at the species level, and sample-level clustering analysis [47]. For each sample, the cleaned reads (after quality control and host removal) were aligned and annotated against the CARD using DIAMOND software with the following parameters: -e 0.001 (i.e., e-value < 1 × 10^−3^) and -i80 (i.e., percent identity > 80%). This allowed for the determination of the relative abundance of potential antibiotic resistance genes in each sample [48].

### 2.10. Metabonomics

Three samples per group were chosen to perform metabolomic analysis. To ensure the quality control of the experiment, QC samples were prepared simultaneously with the sample processing. The QC samples were equal-volume mixtures of the experimental samples, used to balance the chromatography-mass spectrometry system, monitor the instrument status, and evaluate the stability of the system throughout the entire experiment. In addition, blank samples were also set up, mainly for removing background ions.

To begin with, metabolites in feces were extracted. 100 mg of samples ground with liquid nitrogen were placed in EP tubes, and 500 μL of 80% methanol-water solution was added. Vortexed and placed on ice for 5 min, then centrifuged at 15,000× *g* for 20 min in a 4 °C centrifuge. A certain amount of the supernatant was taken and diluted with mass spectrometry-grade water to a methanol content of 53%. Again, perform a 15,000× *g*, 20 min centrifugation at 4 °C. Collect the supernatant and analyze it using Liquid Chromatography-Mass Spectrometry (mass spectrometer: Q Exactive^TM^ HF/Q Exactive^TM^ HF-X, Thermo Fisher, Dreieich, Germany. Chromatograph: Vanquish UHPLC, Thermo Fisher, Germany. Chromatographic column: Hypesil Gold column, 100 × 2.1 mm, 1.9 μm (Thermo Fisher, Dreieich, Germany). Take equal volumes of samples from each experimental sample and mix them together as QC samples. Replace the experimental samples with 53% methanol-water solution and perform the same pre-treatment process. Set the column temperature to 40 °C and the flow rate to 0.2 mL/min. The mobile phase A is 0.1% formic acid, and the mobile phase B is methanol. The chromatographic gradient elution program is shown in Table 2.

A scan range of *m*/*z* 100 to 1500 was configured for the mass spectrometry analysis. The settings for the ESI source are as follows: Spray Voltage: 3.5 kV, Sheath gas flow rate: 35 psi, Auxiliary gas flow rate: 10 L/min, Capillary Temp: 320 °C; S-lens RF level: 60, Aux gas heater temp: 350 °C, Polarity: positive, negative. MS/MS secondary scans are data-dependent scans.

Convert the off-machine data file to mzXML format using ProteoWizard 3.0. First, perform peak extraction and peak quantification using XCMS 4.4.0, align the peaks based on retention time, mass-to-charge ratio, etc., and correct the peak areas for different samples using the first sample as a reference to make the quantification more accurate. Additionally, compare the results with high-quality secondary spectra databases based on the settings of 10 ppm mass deviation and adduct ions, and perform metabolite identification. Subsequently, remove background ions using the blank sample, standardize the original quantitative results according to the formula: Sample original quantitative value/(Sum of sample metabolite quantitative values/Sum of quantitative values of the first sample metabolites), and obtain relative peak areas.

Annotate the identified metabolites using the KEGG database (https://www.genome.jp/kegg/pathway.html, (accessed on 26 May 2025)), HMDB database (https://hmdb.ca/metabolites, (accessed on 26 May 2025)), and LIPIDMaps database (http://www.lipidmaps.org/, (accessed on 26 May 2025)).

### 2.11. Statistical Analysis

Statistical analysis of the experimental data was performed using SPSS 26.0 software. For indicators including endurance capacity, food intake, water intake, gut microbiome characteristics, intestinal inflammatory factors, and gut microbiome α diversity, one-way analysis of variance (ANOVA) coupled with Bonferroni or Tamhane T2 post hoc tests was used to assess the statistical significance of differences between two or more groups. In cases where the data failed to conform to a normal distribution, the Kruskal–Wallis test was applied for statistical analysis of nonparametric data. A *p*-value < 0.05 was considered to indicate statistically significant differences among groups. Permutational multivariate analysis of variance (PERMANOVA) was utilized to analyze the Bray–Curtis dissimilarity. Differences in microbial community structures were examined via principal coordinate analysis (PCoA) based on Bray–Curtis dissimilarity, while variations among fecal samples were evaluated through QIIME2 software(2022.2).

## 3. Results

### 3.1. Body Weight, Water Intake, and Food Intake of Mice

**Figure 2:**
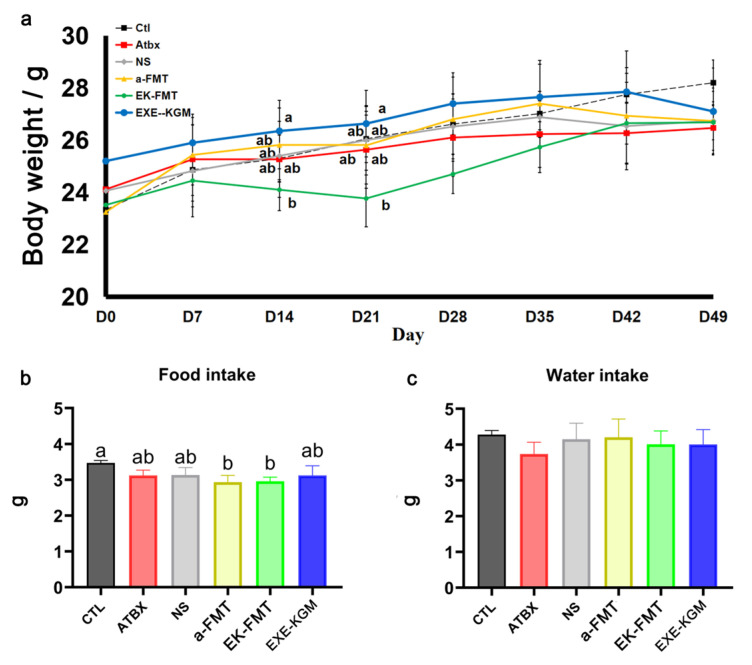
(**a**) The body weight of mice, (**b**) the average daily food intake of mice, (**c**) the average daily water intake of mice. Different numbers indicate different days after antibiotic intervention. Different letters indicate the significant difference among different groups for the same index; ANOVA with Bonferroni or Tamhane’s T2 post hoc tests, or Kruskal–Wallis test. The average of six or five samples, error bars represent the standard deviation at *N* = 6 or 5. CTL: control; ATBX: antibiotic; NS: Normal Saline; a-FMT: Autologous-FMT; EK-FMT: Exercise combined with KGM intervention mice FMT; EXE-KGM: exercise combined with KGM intervention.

As shown in Figure 2a, during the two-week period of FMT intervention (D7-D21), the body weight of mice in the EK-FMT group decreased significantly compared with the EXE-KGM group. One week after the intervention of FMT (D28), the body weight of mice in the EK-FMT group still showed a significant difference compared with the EXE-KGM group. However, with the extension of time, the body weight of the EK-FMT group mice gradually recovered. Two weeks after the intervention of FMT (D35), there was no significant difference in the body weight of the mice in each group. As shown in Figure 2c, there was no significant difference in the average daily water intake of mice.

As shown in Figure S1a,b, the weight and water intake of mice in the EK-Donor group were relatively stable despite some fluctuations. In the formal experiment, the water and food intakes of all groups of mice remained relatively stable. Figure S1c,d shows the change in endurance between the EK-Donor group and the EXE-KGM group. The maximum running distance in the EK-Donor group increased after three weeks of intervention, but the maximum running distance in the EXE-KGM group decreased after antibiotic intervention. The maximum running distance did not increase after three weeks of intervention, even though there was no significant difference in these changes. However, it is still possible to assume that antibiotics affect mice’s endurance, which is consistent with previous studies [29]. As shown in Figure S2e, compared with the EK-Donor group, the average daily water intake of mice in the EXE-KGM group was not significantly different, indicating that there was no significant difference in the intake of KGM between the two groups.

### 3.2. Intestinal Morphology and Intestinal Inflammation in Mice

**Figure 3:**
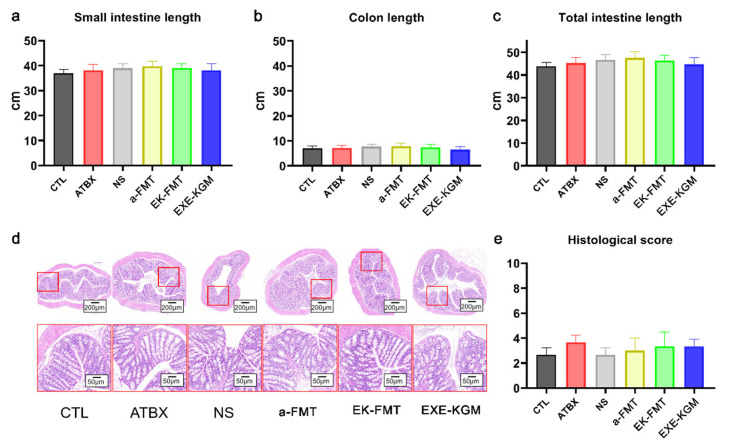
Effects of different intervention methods on intestinal tissues of mice. (**a**) small intestine length, (**b**) colon length, (**c**) Total intestine length, (**d**) HE staining map, (**e**) intestinal histological score. ANOVA with Bonferroni or Tamhane’s T2 post hoc test, or Kruskal–Wallis test. Average of six or five samples, error bars represent standard deviation at *N* = 6 or 5 (**a**–**c**), *N* = 3 (**d**,**e**). CTL: control; ATBX: antibiotic; NS: Normal Saline; a-FMT: Autologous-FMT; EK-FMT: Exercise combined with KGM intervention mice FMT; EXE-KGM: exercise combined with KGM intervention.

As shown in Figure 3, there were no significant differences observed in the lengths of the small intestine, colon, and whole intestine of mice among groups with different intervention methods. There was no significant difference in colon HE staining and histological scores across all groups.

**Figure 4:**
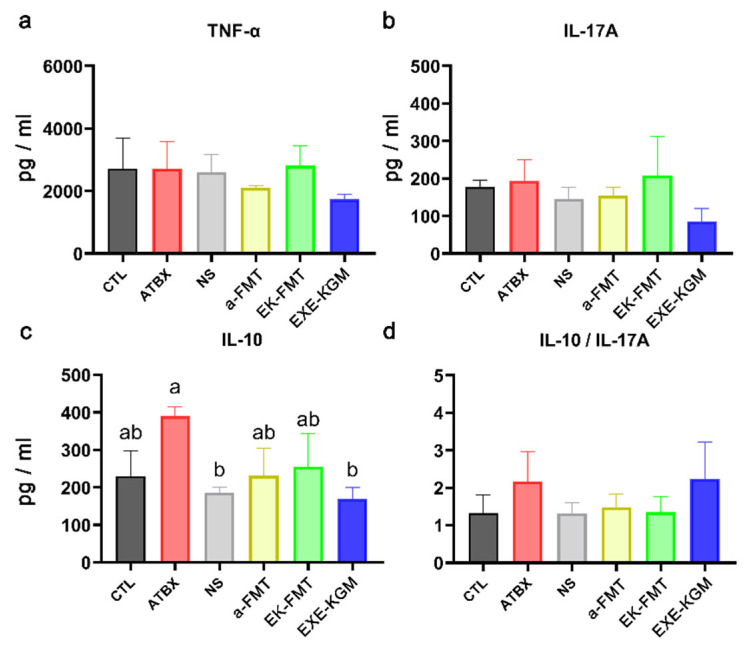
Effects of different intervention methods on the inflammation of mice. (**a**) TNF-α, (**b**) IL-17A, (**c**) IL-10, (**d**) IL-10/IL-17A. Different letters indicate the significant difference among different groups for the same index; ANOVA with Bonferroni or Tamhane’s T2 post hoc test, or Kruskal–Wallis test. Average of three samples, error bars represent standard deviation at *N* = 3. CTL: control; ATBX: antibiotic; NS: Normal Saline; a-FMT: Autologous-FMT; EK-FMT: Exercise combined with KGM intervention mice FMT; EXE-KGM: exercise combined with KGM intervention.

Figure 4 illustrates the inflammatory factors present in colon tissues. Although a decreasing trend was observed in the TNF-α levels for both the a-FMT and EXE-KGM groups, no statistically significant differences were found in either TNF-α (Figure 4a) or IL-17A (Figure 4b) among the groups. For IL-10 (Figure 4c), the ATBX group exhibited a marginal increase, while both the NS and EXE-KGM groups showed a significant decrease compared to the ATBX group. However, no statistical differences were noted in the ratio of IL-10 to IL-17A (Figure 4d).

### 3.3. Effects of Different Interventions on Gut Microbiome in Mice

**Figure 5:**
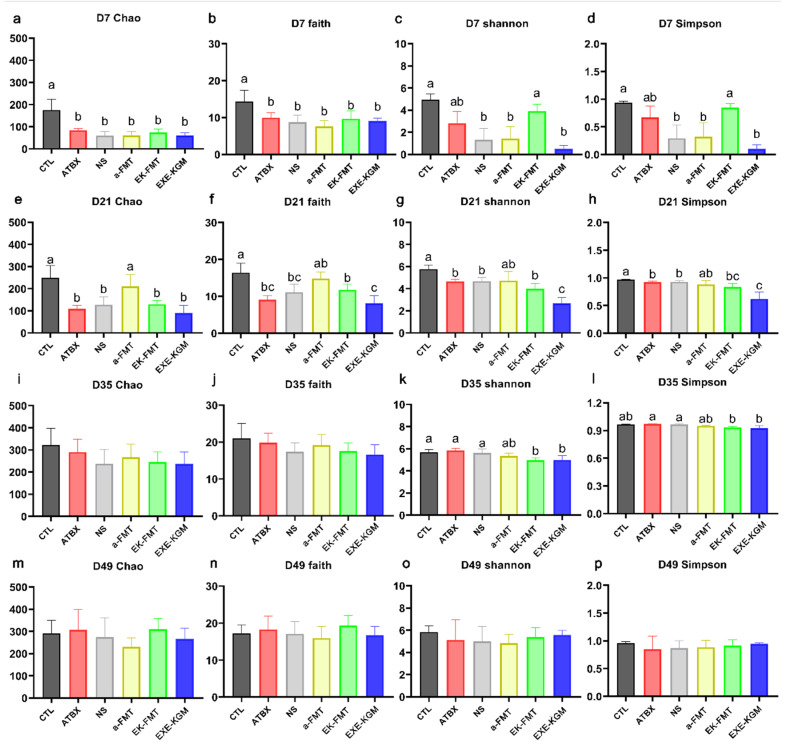
Effect of different time points on α-diversity of the gut microbiome in mice. (**a**) D7 Chao1, (**b**) D7 faith_pd, (**c**) D7 shannon, (**d**) D7 Simpson (**e**) D21 Chao1, (**f**) D21 faith_pd, (**g**) D21 shannon, (**h**) D21 Simpson (**i**) D35 Chao1, (**j**) D35 faith_pd, (**k**) D35 shannon, (**l**) D35 Simpson (**m**) D49 Chao1, (**n**) D49 faith_pd, (**o**) D49 shannon, (**p**) D49 Simpson. Different numbers indicate different days after antibiotic intervention. Different letters indicate the significant difference among different groups for the same index; ANOVA with Bonferroni or Tamhane’s T2 post hoc test, or Kruskal–Wallis test. The average of six or five samples, error bars represent the standard deviation at *N* = 6 or 5. CTL: control; ATBX: antibiotic; NS: Normal Saline; a-FMT: Autologous-FMT; EK-FMT: Exercise combined with KGM intervention mice FMT; EXE-KGM: exercise combined with KGM intervention.

Depicted in Figure 5a–d (D7), the Chao1 and Faith_pd indices of the five groups after antibiotic administration were significantly lower than those in the CTL group. The Shannon and Simpson indices in the EK-FMT group recovered to levels analogous to those in the CTL group and were notably higher than those detected in the NS, a-FMT, and EXE-KGM groups. However, upon completion of FMT intervention (Figure 5e–h), only the a-FMT group showed no statistically significant differences in Chao1, Faith_pd, Shannon, and Simpson indices when compared with the CTL group. Meanwhile, the α diversity of the EXE-KGM group was markedly lower than that of the other five groups. Illustrated in Figure 5i–l, two weeks after FMT intervention (D35), the Shannon index in both the EK-FMT and EXE-KGM groups was significantly lower than that in the CTL group. As presented in Figure 5m–p, no significant intergroup differences were observed at the final experimental endpoint.

**Figure 6:**
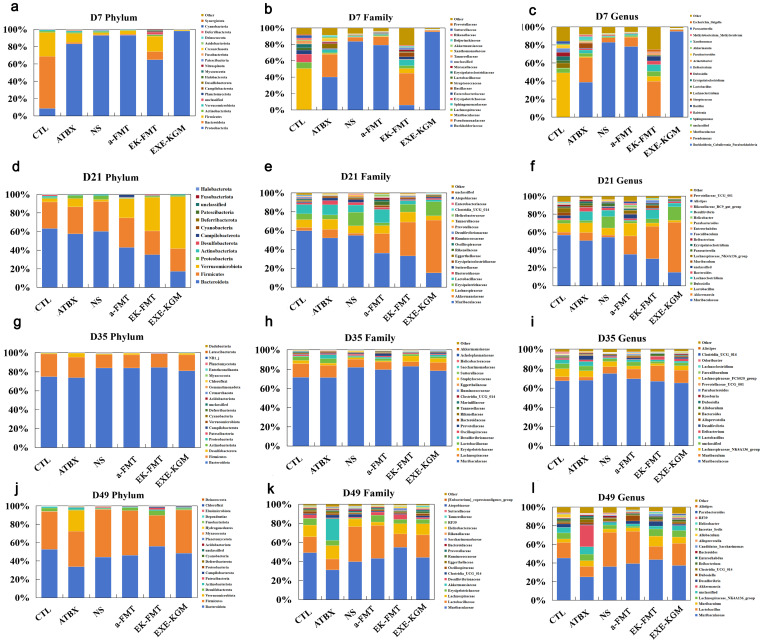
Effects of different intervention methods on fecal microbial composition of mice: (**a**) D7 at phylum level, (**b**) D7 at family level, (**c**) D7 at genus level. (**d**) D21 at phylum level, (**e**) D21 at family level, (**f**) D21 at genus level, (**g**) D35 at phylum level, (**h**) D35 at family level, (**i**) D35 at genus level. (**j**) D49 at the phylum level, (**k**) D49 at the family level, (**l**) D49 at the genus level. The average of six or five samples, error bars represent the standard deviation at *N* = 6 or 5. CTL: control; CTL: control; ATBX: antibiotic; NS: Normal Saline; a-FMT: Autologous-FMT; EK-FMT: Exercise combined with KGM intervention mice FMT; EXE-KGM: exercise combined with KGM intervention.

As shown in Figure 6a–c, after antibiotic intervention (D7), antibiotics generally exerted a significant impact on the gut microbiome abundance in mice. Depicted in Figure 6d–f, following FMT intervention or exercise combined with KGM intervention (D21), the gut microbiome abundance in mice from each group underwent substantial changes. At the phylum level (Figure 6d), in comparison to the CTL group, Bacteroidota was markedly reduced in the EXE-KGM, a-FMT, and EK-FMT groups, while Verrucomicrobiota was significantly elevated, and Deferribacterota was notably diminished. In the ATBX and NS groups, Proteobacteria were significantly higher, and Deferribacterota was markedly lower compared with the CTL group. At the family level (Figure 6e), Muribaculaceae was significantly reduced, and Akkermansiaceae was notably increased in the EXE-KGM and EK-FMT groups when compared with the CTL group. Muribaculaceae also exhibited a significant decrease in the EK-FMT and a-FMT groups relative to the CTL group. At the genus level (Figure 6f), *Muribaculaceae* and *Lachnospiraceae_NK4A136_group* were significantly reduced in the EXE-KGM and EK-FMT groups compared with the CTL group, while *Muribaculaceae* was notably diminished in the EK-FMT and a-FMT groups. Additionally, *Lachnoclostridium* was significantly higher in both the ATBX and NS groups relative to the CTL group. Overall, after FMT intervention or exercise combined with KGM intervention (D21), the gut microbiome abundance in the EXE-KGM group still differed significantly from that in the CTL group, whereas the gut microbiome abundance in the a-FMT group was more similar to that in the CTL group.

Illustrated in Figure 6g–i, two weeks after the termination of FMT or exercise combined with KGM intervention (D35), the gut microbiome abundance in each group of mice also changed significantly. At the family level (Figure 6h), Tannerellaceae and Marinifilaceae were significantly reduced in the EXE-KGM, EK-FMT, and a-FMT groups compared with the CTL group. Marinifilaceae was also notably diminished in the ATBX and NS groups relative to the CTL group. At the genus level (Figure 6i), *Parabacteroides* was significantly increased, while *Odoribacter* was markedly decreased in the EXE-KGM, EK-FMT, and a-FMT groups compared with the CTL group. Furthermore, *Odoribacter* was significantly lower in the ATBX and NS groups relative to the CTL group. Therefore, two weeks after intervention, significant differences in gut microbiome abundance were observed between the EXE-KGM, EK-FMT, a-FMT groups and the CTL group.

As presented in Figure 6j–l, at the experimental endpoint (D49), with the extension of recovery time, the gut microbiome abundance in mice underwent further alterations. At the phylum level (Figure 6j), only the ATBX group showed a significant increase in Verrucomicrobiota compared with the CTL group. At the family level (Figure 6k), the Eubacterium_coprostanoligenes_group was significantly elevated in the ATBX group relative to the CTL group. At the genus level (Figure 6l), *Muribaculum* was significantly increased in the EK-FMT group compared with the CTL group, while *Allobaculum* was notably decreased in the ATBX and EXE-KGM groups relative to the CTL group. In summary, at the experimental endpoint, the ATBX group exhibited the most pronounced variations in the abundance of specific gut microbiome compared with the CTL group. The relative abundances of gut bacteria that showed significant changes at different time points are summarized in Tables S4–S6.

**Figure 7:**
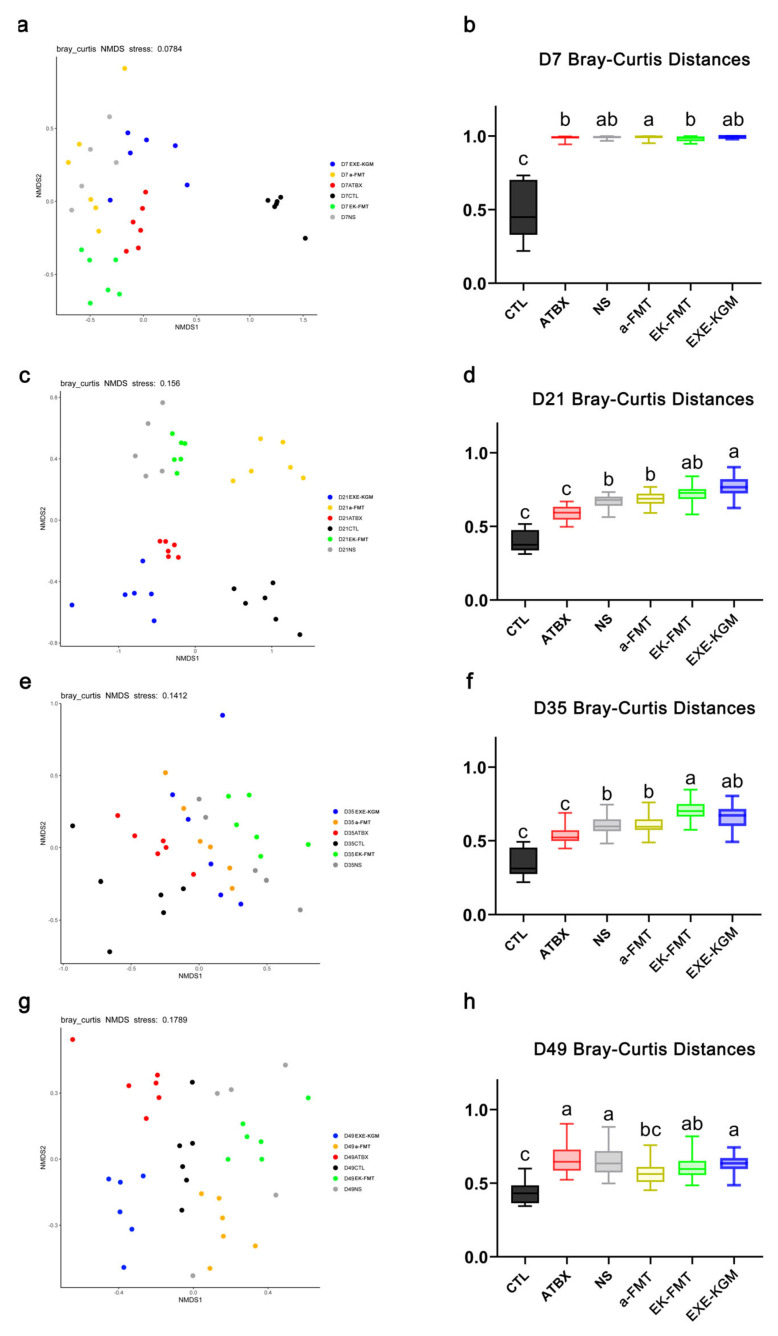
At different time points, the NMDS analysis based on Bray–Curtis distance and the comparison of Bray–Curtis distances (**a**) D7-PCoA, (**b**) D7-Bray–Curtis distances from five groups to CTL group, (**c**) D21-PCoA, (**d**) D21-Bray–Curtis distances from five groups to CTL group, (**e**) D35-PCoA, (**f**) D35-Bray–Curtis distances from five groups to CTL group, (**g**) D49-PCoA, (**h**) D49-Bray–Curtis distances from five groups to CTL group. Different numbers indicate different days after antibiotic intervention Different letters indicate the significant difference among different groups for the same index; ANOVA with Bonferroni or Tamhane’s T2 post hoc test, or Kruskal–Wallis test. The average of six or five samples, error bars represent the standard deviation at *N* = 6 or 5. CTL: control; ATBX: antibiotic; NS: Normal Saline; a-FMT: Autologous-FMT; EK-FMT: Exercise combined with KGM intervention mice FMT; EXE-KGM: exercise combined with KGM intervention.

Depicted in Figure 7, after one week of antibiotic intervention (Figure 7a,b), antibiotic intervention resulted in a significant increase in the Bray–Curtis distances between the gut microbiome of mice and that of the CTL group, indicating a substantial microbial community shift induced by antibiotic disturbance. After two weeks of FMT or exercise combined with KGM intervention (D21, Figure 7c,d), the Bray–Curtis distances of gut microbiome between the ATBX and CTL groups were found to be closer. In contrast, the microbial community structure in the EK-FMT and EXE-KGM groups displayed a greater divergence from that of the CTL group. Following a two-week recovery period (D35, Figure 7e,f), the Bray–Curtis distances between the gut microbiome in the ATBX and CTL groups remained similar. Conversely, the Bray–Curtis distances between the EK-FMT group and the CTL group were significantly greater. At the experimental endpoint (Figure 7g,h), the gut microbiome of the a-FMT group showed the closest Bray–Curtis distance to that of the CTL group.

### 3.4. The Production of Individual and Total SCFAs

**Figure 8:**
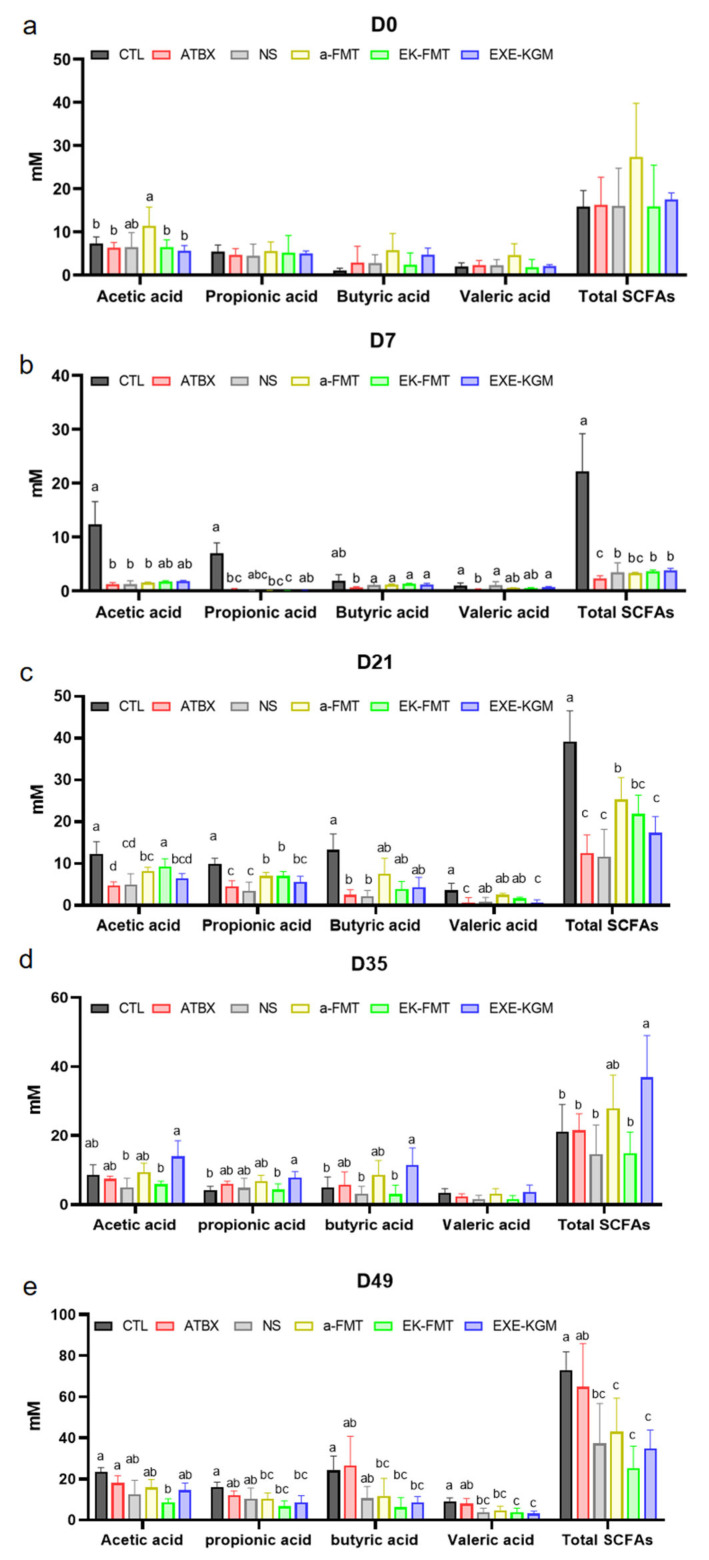
Concentrations of various short-chain fatty acids in feces at different time points: (**a**) D0; (**b**) D7; (**c**) D21; (**d**) D35; (**e**) D49. Different numbers indicate different days after antibiotic intervention. Different letters indicate the significant difference among different groups for the same index; ANOVA with Bonferroni or Tamhane’s T2 post hoc test, or Kruskal–Wallis test. The average of six or five samples, error bars represent standard deviation at *N* = 6 or 5. CTL: control; ATBX: antibiotic; NS: Normal Saline; a-FMT: Autologous-FMT; EK-FMT: Exercise combined with KGM intervention mice FMT; EXE-KGM: exercise combined with KGM intervention.

Figure 8 shows the change in individual and total SCFAs concentration in feces.

As shown in Figure 8a, at the baseline, there were no significant differences in fecal SCFAs among the groups except for acetic acid. The difference in acetic acid might be due to the two-week fecal collection before the experiment for the a-FMT group mice, which slightly affected the SCFAs in the mice’s feces. As shown in Figure 8b, after antibiotic intervention, compared with the CTL group, SCFAs in the other five groups of mice showed a decreasing trend. Among them, the total SCFA concentration in the antibiotic-intervention mice was significantly lower than that in the CTL group, demonstrating that antibiotic intervention significantly affected the ability of the gut microbiome to produce SCFAs in mice.

At the end of FMT (D21, Figure 8c), the concentrations of individual and total SCFA in the ATBX group were all significantly decreased compared with the CTL group. The concentrations of individual and total SCFAs in the EXE-KGM group were comparable with those of the ATBX group. In contrast, the concentrations of acetic acid, propionic acid, and total SCFAs in the a-FMT and EK-FMT groups were significantly increased compared with the ATBX group. The a-FMT and EK-FMT groups maintained concentrations comparable with CTL in the concentrations of butyric acid and valeric acid, and there was also no significant decrease in the concentration of acetic acid in the EK-FMT group.

As shown in Figure 8d, two weeks after the intervention of FMT or exercise combined with KGM intervention (D35), compared with the CTL group, only the EXE-KGM group had significantly increased concentrations of propionic acid, butyric acid, and total SCFAs. Furthermore, the individual and SCFA levels returned to comparable levels with those of the CTL group. Additionally, no significant differences were observed in either individual or total SCFA levels between the ATBX group and all intervention groups.

As illustrated in Figure 8e, at the endpoint of the experiment (D49), both individual and total SCFA concentrations in the ATBX group remained comparable to those observed in the CTL group. In contrast, valeric acid and total SCFA concentrations were significantly reduced in the EK-FMT and EXE-KGM groups when compared with both the CTL and ATBX groups. Additionally, a decreasing trend was also noted in the NS and a-FMT groups. The levels of propionic acid, butyric acid, valeric acid, and total SCFAs in the a-FMT, EK-FMT, and EXE-KGM groups were markedly lower than those found in the CTL group. Furthermore, acetic acid concentration within the EK-FMT group was significantly diminished relative to that of the CTL group.

Figure S2 shows the changes in different SCFAs in each group over time. The results indicate that after antibiotic intervention, compared with the CTL group, different SCFAs in each group decreased significantly. After two weeks of intervention, the different intervention methods, or self-recovery (ATBX group), all returned to normal levels.

Additionally, the correlation analysis of the gut microbiome and SCFAs at different time points in mice were conducted. It was found that at the phylum level, at D7 (Figure S3a), Proteobacteria has a significant negative correlation with acetic acid. Bacteroidota, Firmicutes, Campilobacterota, and Deferribacterota have a significant positive correlation with acetic acid. Verrucomicrobiota, Campilobacterota, Desulfobacterota, and Deferribacterota have a significant positive correlation with propionic acid, while Bacteroidota, Campilobacterota, Deferribacterota, and total SCFAs have a significant positive correlation. Cyanobacteria has a significant positive correlation with all types of SCFAs. At D21 (Figure S3b), Proteobacteria were significantly negatively correlated with the concentrations of various SCFAs in feces, while Cyanobacteria, Deferribacterota, and Patescibacteria were significantly positively correlated with the concentrations of various SCFAs in feces. At D35 (Figure S3c), Patescibacteria were significantly negatively correlated with acetic acid, propionic acid, butyric acid, and total SCFAs; Cyanobacteria were negatively correlated with acetic acid, propionic acid, and valeric acid. At D49 (Figure S3d), Verrucomicrobiota was significantly positively correlated with acetic acid, butyric acid, valeric acid, and total SCFAs; conversely, Campilobacterota was significantly negatively correlated with various SCFAs in feces.

At the family level, at D7 (Figure S4a), Pseudomonadaceae, Beijerinckiaceae, and various SCFAs were significantly negatively correlated. Erysipelotrichaceae, Sutterellaceae and various SCFAs are significantly positively correlated. The Sphingomonadaceae family has a significant negative correlation with acetic acid, propionic acid, butyric acid, and total SCFAs. Rikenellaceae is significantly positively correlated with acetic acid, propionic acid, butyric acid, and total SCFAs. At D21 (Figure S4b), Rikenellaceae, Oscillospiraceae, and Ruminococcaceae were significantly positively correlated with various SCFAs in feces; Enterobacteriaceae and Sutterellaceae were significantly negatively correlated with various SCFAs in feces. At D35 (Figure S4c), Prevotellaceae was significantly positively correlated with butyric acid; Saccharimonadaceae and Helicobacteraceae were significantly negatively correlated with various SCFAs in feces. At D49 (Figure S4d), Helicobacteraceae was also significantly negatively correlated with various SCFAs in feces.

At the genus level, at D7 (Figure S5a), *Pseudomonas*, *Methylobacterium_Methylorubrum* are significantly negatively correlated with various SCFAs. *Lachnoclostridium*, *Erysipelatoclostridium*, and *Parasutterella* are significantly positively correlated with various SCFAs. *Dubosiella*, *Lleibacterium*, and *Parabacteroides* are significantly positively correlated with acetic acid, propionic acid, and total SCFAs. *Sphingomonas* and *Ralstonia* are significantly negatively correlated with acetic acid, propionic acid, butyric acid, and total SCFAs. At D21 (Figure S5b), *Lachnospiraceae_NK4A136_group*, *Alistipes*, *Prevotellaceae_UCG_001* were significantly positively correlated with various SCFAs in feces; *Lachnoclostridium*, *Parasutterella* were significantly negatively correlated with various SCFAs in feces. At D35 (Figure S5c), *Alloprevotella* was significantly positively correlated with propionic acid; *Allobaculum* was significantly negatively correlated with propionic acid. At D49 (Figure S5d), *Helicobacter* was significantly negatively correlated with various SCFAs in feces, and *Enterorhabdus* was significantly negatively correlated with butyric acid, valeric acid, and total SCFAs.

Overall, Proteobacteria, Helicobacteraceae, and Helicobacter, as potential or conditional pathogenic bacteria [49], show significant negative correlations with various SCFAs. On the contrary, Ruminococcaceae, Lachnospiraceae, Parabacteroides, and Verrucomicrobiota show significant positive correlations with various SCFAs. Ruminococcaceae and Lachnospiraceae can ferment indigestible dietary fibers or polysaccharides. Parabacteroides is involved in carbohydrate metabolism and the production of SCFAs such as acetic acid.

### 3.5. Metagenomics

Metagenomic analysis indicates that the ATBX group primarily affected core genetic information processing and basic metabolic maintenance, while the a-FMT and EXE-KGM group further drove coordinated remodeling of amino acid metabolism, carbohydrate utilization, nucleotide biosynthesis, energy production, and cell wall construction. Among them, EXE-KGM group showed a more integrated metabolic shift linking enhanced energy generation and lipid remodeling to protein synthesis and structural adaptation, suggesting a progressive recovery and functional reinforcement of the gut microbiome across the intervention groups.

**Figure 9:**
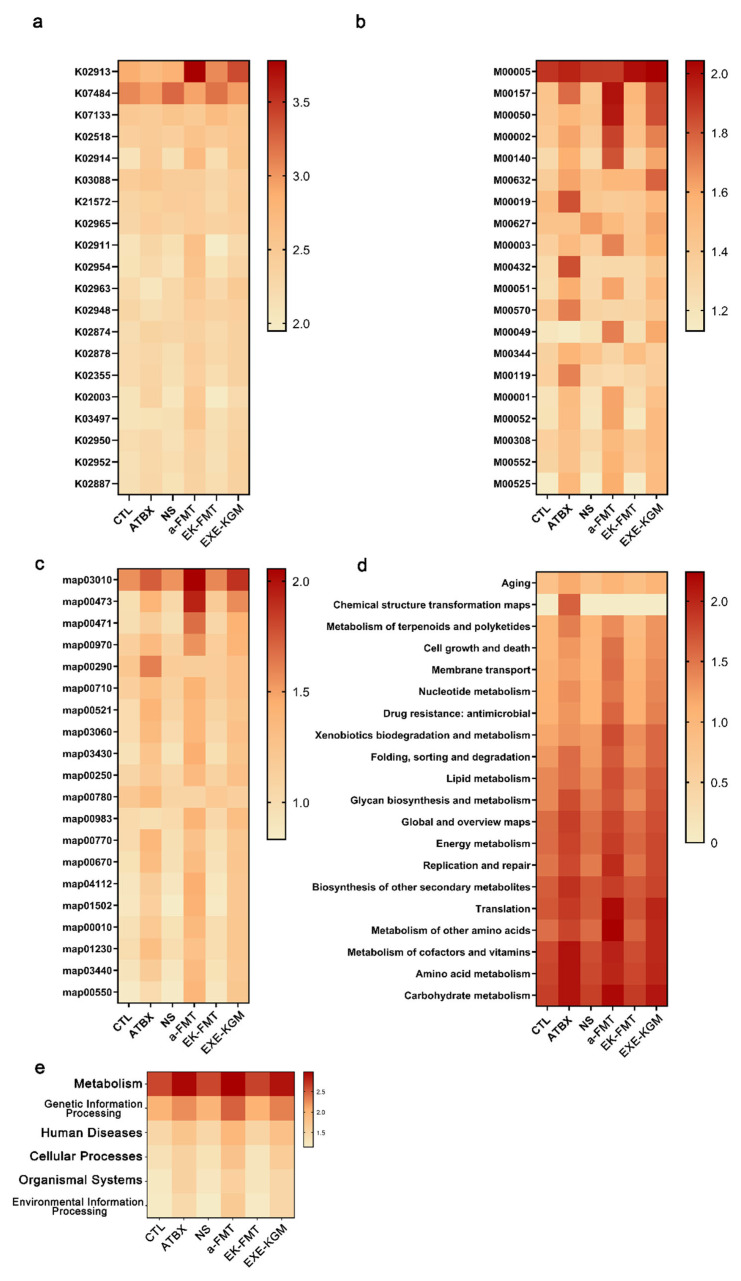
Heatmap showing the effects of different intervention methods on the KEGG metabolic pathways in mice at day 49. (**a**) KO, (**b**) KEGG Module, (**c**) KEGG Pathway, (**d**) KEGG Pathway Level1, (**e**) KEGG Pathway Level2. Samples (*N* = 3) were colored by treatment. CTL: control; ATBX: antibiotic; NS: Normal Saline; a-FMT: Autologous-FMT; EK-FMT: Exercise combined with KGM intervention mice FMT; EXE-KGM: exercise combined with KGM intervention.

Specifically, according to the analysis of the KEGG ORTHOLOGY database (Figure 9a), the EXE-KGM group was enriched for map00473, while the a-FMT group was enriched for map00473 and map00471. Map00473 corresponds to D-alanine metabolism, which is closely involved in bacterial amino acid interconversion and peptidoglycan synthesis, playing a critical role in cell wall formation and microbiota growth. Map00471 corresponds to D-glutamine and D-glutamate metabolism, which is essential for nitrogen metabolism, amino acid homeostasis, and cell wall biosynthesis in bacteria. According to the analysis of KEGG pathway level 1 (Figure 9d), the EXE-KGM, a-FMT, and ATBX groups were all enriched for “Genetic Information Processing”. According to the analysis of KEGG pathway level 2 (Figure 9e), the EXE-KGM, a-FMT, and ATBX groups were enriched for “Metabolism of other amino acids,” “Replication and repair”, “Glycan biosynthesis and metabolism”, “Lipid metabolism,” and “Folding, sorting and degradation” reflecting coordinated modulation of amino acid turnover, genome stability maintenance, cell surface polysaccharide synthesis, lipid utilization, and protein quality control systems. Both the a-FMT group and the EXE-KGM group were significantly enriched for K02913. According to the KEGG modules analysis (Figure 9b), the EXE-KGM, a-FMT, and ATBX groups were all enriched for M00005 and M00157. M00005 represents the oxidative phase of the pentose phosphate pathway, which generates NADPH and ribose-5-phosphate for reductive biosynthesis and nucleotide production, while M00157 corresponds to the F-type ATP synthase module responsible for ATP generation via oxidative phosphorylation. Additionally, the EXE-KGM group was enriched for M00632 and M00050, which correspond to galactose degradation and de novo inosine monophosphate (IMP) biosynthesis, indicating enhanced carbohydrate utilization and purine nucleotide synthesis capacity The a-FMT group was enriched for M00050, M00002, M00140, and M00049, representing IMP biosynthesis, core glycolysis, one-carbon unit interconversion, and adenine ribonucleotide biosynthesis, respectively, suggesting coordinated regulation of central carbon metabolism and nucleotide biosynthetic pathways. According to the KEGG pathways analysis (Figure 9c), the EXE-KGM, a-FMT, and ATBX groups were all enriched for map03010.

**Figure 10:**
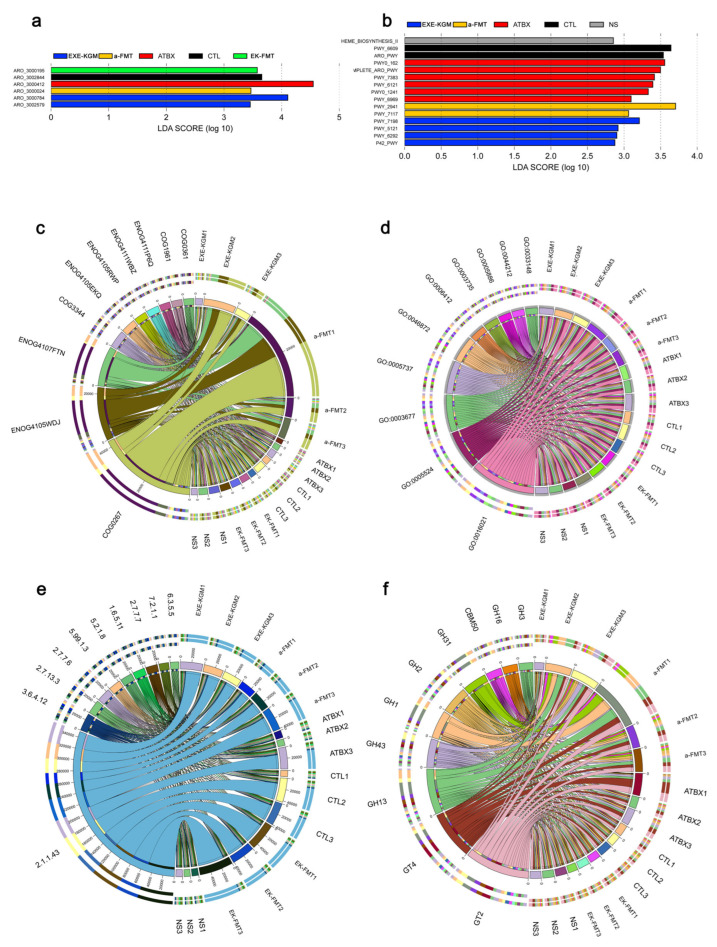
The effects of different intervention methods on the (**a**) ARO-LDA bar chart, (**b**) Metacyc-LDA bar chart, (**c**) EggNog-Circos chart, (**d**) GO Circos chart, (**e**) EC enzymes Circos chart, (**f**) CAZy-Circos chart in the feces on day 49. Samples (*N* = 3) were colored by treatment. CTL: control; ATBX: antibiotic; NS: Normal Saline; a-FMT: Autologous-FMT; EK-FMT: Exercise combined with KGM intervention mice FMT; EXE-KGM: exercise combined with KGM intervention.

According to the analysis of the MetaCyc database (Figure 10b), the a-FMT group was enriched with PWY_2941 and PWY_7117, which correspond to lysine biosynthesis via the diaminopimelate pathway and pyrimidine ribonucleotide salvage, respectively, reflecting enhanced amino acid biosynthesis and nucleotide recycling processes. The EXE-KGM group was enriched with PWY_7198, PWY_5121, PWY_6292, and P42_PWY, which are mainly involved in coenzyme A biosynthesis, branched-chain amino acid degradation, fatty acid β-oxidation, and polysaccharide precursor biosynthesis, indicating increased metabolic flexibility in energy, lipid, and carbohydrate metabolism. According to the analysis of the EggNOG database (Figure 10c), the EK-FMT group and the a-FMT group were enriched for ENOG4107FTN, which is generally associated with transcriptional regulation and stress response–related protein functions, suggesting altered microbial adaptability. According to the analysis of the GO database (Figure 10d), the a-FMT, EK-FMT, and EXE-KGM groups enriched GO:0044212, GO:0045944, GO:0044666, GO:0043627, GO:0042800, GO:0008284, and GO:0033148. According to the analysis of the EC enzyme database (Figure 10e), the EK-FMT and EXE-KGM groups enriched EC 2.1.1.43, which corresponds to phosphatidylethanolamine N-methyltransferase, an enzyme involved in phospholipid methylation and membrane lipid remodeling. According to the analysis of the CAZy database (Figure 10f), the a-FMT and EXE-KGM groups were enriched for GH13, which mainly includes α-amylase family enzymes involved in starch and glycogen degradation. The a-FMT and ATBX groups enriched GT2 and GT4, which are glycosyltransferase families associated with polysaccharide and cell wall biosynthesis. The a-FMT group enriched GT113, GT32, GH1, GH31, CBM32, and GH73, indicating enhanced capacities for oligosaccharide synthesis, glycosidic bond hydrolysis, carbohydrate binding, and peptidoglycan remodeling.

### 3.6. Metabolomics

As shown in Figure S6, the PCA-3D plot (Figure S6d) indicates that the metabolic composition structures of all samples are highly similar, and there are no significant structural differences among the groups. Figure S6c shows the correction effect of each metabolite after adjusting for signal drift using the QC-RFSC algorithm. After correction, the QC samples cluster closely on the PCA plot, and the correction effect is good, proving that the data obtained from the metabolomics analysis are repeatable and accurate (Figure S6c).

**Figure 11:**
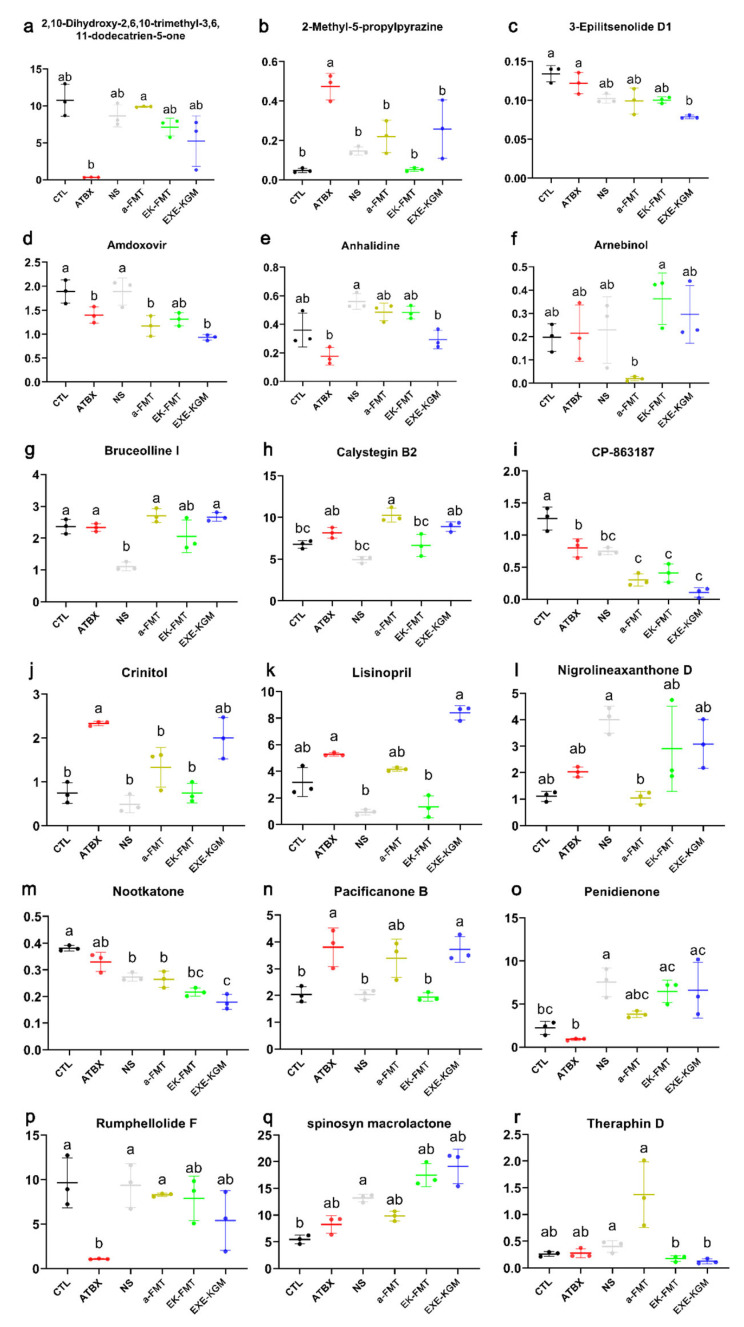
Representative (those with the smallest *p*-values) differentially expressed metabolites. (**a**) 2,10-Dihydroxy-2,6,10-trimethyl-3,6,11, (**b**) 2-Methyl-5-propylpyrazine, (**c**) 3-Epilitsenolide D1, (**d**) Amdoxovir, (**e**) Anhalidine, (**f**) Arnebinol, (**g**) Bruceolline I, (**h**) Calystegin B2, (**i**) CP-863187, (**j**) Crinitol, (**k**) Lisinopril, (**l**) Nigrolineaxanthone D, (**m**) Nootkatone, (**n**) Pacificanone B, (**o**) Penidienone, (**p**) Rumphellolide F, (**q**) spinosyn macrolactone, (**r**) Theraphin D. Different letters indicate the significant difference among different groups for the same index; ANOVA with Bonferroni or Tamhane’s T2 post hoc test or Kruskal–Wallis test. Average of three samples, error bars represent standard deviation at *N* = 3. CTL: control; ATBX: antibiotic; NS: Normal Saline; a-FMT: Autologous-FMT; EK-FMT: Exercise combined with KGM intervention mice FMT; EXE-KGM: exercise combined with KGM intervention.

**Figure 12:**
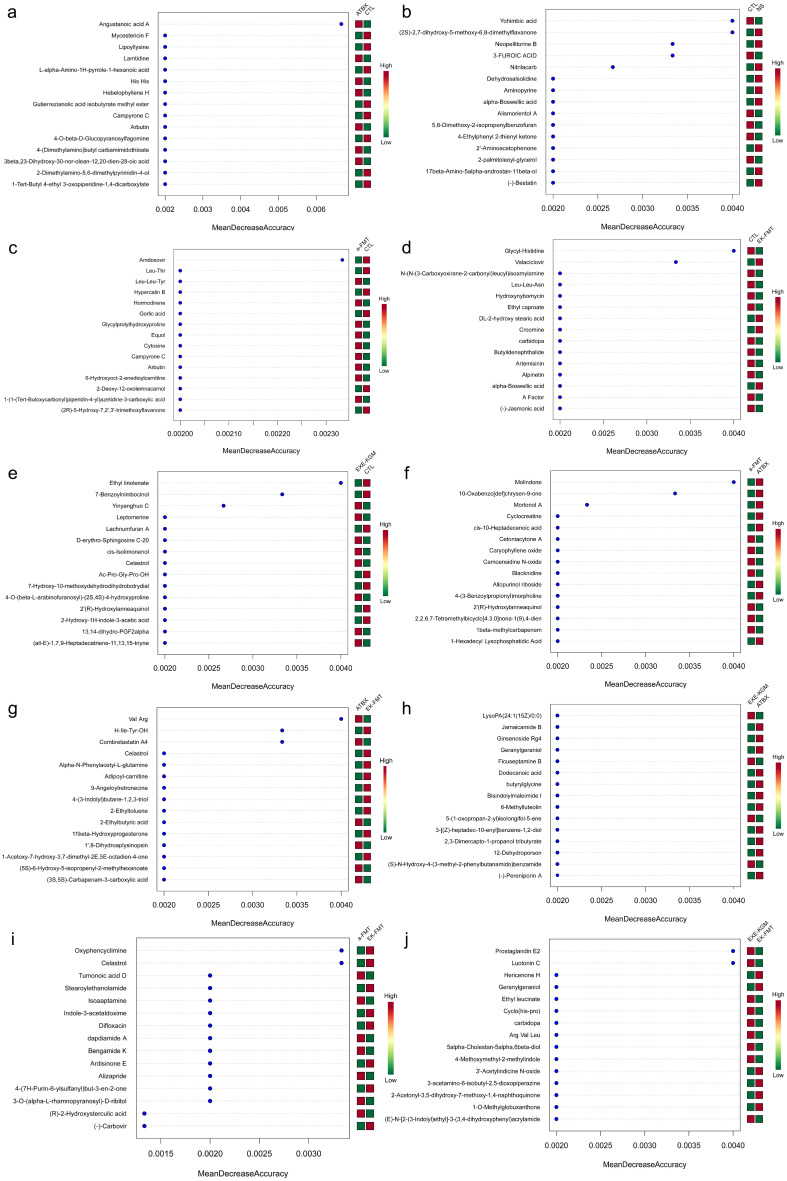
Key metabolites in the random forest after comparison among different groups. (**a**) CTL with ATBX, (**b**) CTL with NS, (**c**) CTL with a-FMT, (**d**) CTL with EK-FMT, (**e**) CTL with EXE-KGM, (**f**) ATBX with a-FMT, (**g**) ATBX with EK-FMT, (**h**) ATBX with EXE-KGM, (**i**) a-FMT with EK-FMT, (**j**) EK-FMT with EXE-KGM. Samples (*N* = 3) were colored by treatment. CTL: control; ATBX: antibiotic; NS: Normal Saline; a-FMT: Autologous-FMT; EK-FMT: Exercise combined with KGM intervention mice FMT; EXE-KGM: exercise combined with KGM intervention.

Although the top 20 most abundant metabolites showed no significant differences in concentration among the groups (Figure S6a), and there were also no notable differences in their biological functions within metabolic pathways (Figure S6b), it was observed that variations existed in 18 metabolites across the different groups (as shown in Figure 11). Compared with the CTL group, the disruption of antibiotics induced a significant increase of 2-Methyl-5-propylpyrazine (Figure 11b), crinitol (Figure 11j), pacificanone B (Figure 11n), while a decrease in amdoxovir and CP-863187 (Figure 11d and Figure 12i). The 2-Methyl-5-propylpyrazine, crinitol, and pacificanone B are mainly microbial-derived secondary metabolites, which are commonly associated with gut microbial amino acid metabolism and terpenoid-related metabolic pathways. These metabolites are typically produced during microbial fermentation processes, and their altered abundance reflects changes in gut microbial metabolic activity following antibiotic-induced microbiota disruption.

Compared with the ATBX, the a-FMT, EK-FMT, and EXE-KGM groups showed a further significant decrease in CP-863187 (Figure 11i). CP-863187 is a microbiota-associated metabolite involved in microbial xenobiotic and drug-like compound metabolism, and its decreased abundance indicates alterations in microbial-mediated biotransformation pathways following different interventions.

Compared with the CTL and ATBX groups, the content of 3-Epilitsenolide D1 in the EXE-KGM group significantly decreased (Figure 11c). 3-Epilitsenolide D1 is considered a microbial-associated lactone compound related to lipid-associated secondary metabolic pathways, and its reduction suggests a specific modulation of microbial lipid-related metabolism in response to the EXE-KGM intervention.

Compared with the EK-FMT group, the content of Arnebinol in the a-FMT group significantly decreased (Figure 11f). Arnebinol is a terpenoid-derived microbial metabolite associated with microbial isoprenoid and secondary metabolite biosynthesis, and its altered levels further indicate that different fecal microbiota transplantation strategies resulted in distinct microbial secondary metabolic profiles.

In most comparisons, only a few metabolites showed differences between the two groups. Notably, NS induced changes in five metabolites, including (2S)-2,7-dihydroxy-5-methoxy-6,8-dimethylflavanone, neopellitorine B, and nitrilacarb. Similarly, a-FMT led to a decrease in three metabolites compared with the ATBX group, namely molindone, 10-Oxabenzo[def]chrysen-9-one, and nortonol A, whereas the increase in H-lle-Tyr-OH induced by EK-FMT was more pronounced than that observed in the ATBX group. These differential metabolites mainly belong to microbial-associated secondary metabolites and amino acid-related metabolites, involving flavonoid-related metabolism, alkaloid and lipid-derived secondary metabolic pathways, as well as peptide and amino acid metabolism, reflecting selective alterations in gut microbial metabolic outputs under different intervention conditions.

When comparing the CTL group with the ATBX group, it was found that the ATBX group significantly enriched angustanoic acid A (Figure 12a); when comparing the CTL group with the NS group, it was found that CTL significantly enriched yohimbic acid, 3-FUROIC ACID, and NS significantly enriched (2S)-2,7-dihydroxy-5-methoxy-6,8-dimethylflavanone, neopellitorine B, and nitrilacarb (Figure 12b). When comparing the CTL group with the a-FMT group, it was found that the CTL significantly enriched amdoxovir (Figure 12c). When comparing the CTL group with the EK-FMT group, it was found that the CTL group significantly enriched GlycyI-Histidine, and the EK-FMT group significantly enriched valaciclovir (Figure 12d). When comparing the CTL group with the EXE-KGM group, it was found that the CTL group significantly enriched ethyl linolenate, 7-Benzoyinimbocinol, and the EXE-KGM group significantly enriched yinyanghuo C (Figure 12e). When comparing the ATBX group with the a-FMT group, it was found that the ATBX group enriched molindone, 10-Oxabenzo[def]chrysen-9-one, and mortonol A (Figure 12f). When comparing the ATBX group with the EK-FMT group, it was found that the ATBX group enriched val Arg, combretastatin A4, and the EK-FMT group enriched H-Ile-Tyr-OH (Figure 12g). When comparing the a-FMT group with the EK-FMT group, it was found that the EK-FMT group enriched oxyphencyclimine and celastrol (Figure 12i). When comparing the EXE-KGM group with the EK-FMT group, it was found that the EXE-KGM group enriched prostaglandin E2 and luotonin C (Figure 12j).

**Figure 13:**
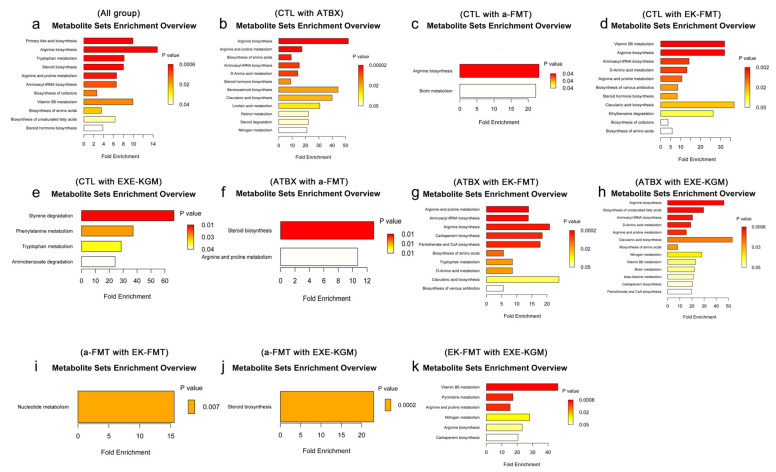
ORA enrichment analysis identified the key biological pathways that played a significant role in the comparison of different groups. (**a**) all group, (**b**) CTL with ATBX, (**c**) CTL with a-FMT, (**d**) CTL with EK-FMT, (**e**) CTL with EXE-KGM, (**f**) ATBX with a-FMT, (**g**) ATBX with EK-FMT, (**h**) ATBX with EXE-KGM, (**i**) a-FMT with EK-FMT, (**j**) a-FMT with EXE-KGM, (**k**) EK-FMT with EXE-KGM. Samples (*N* = 3) were colored by treatment. CTL: control; ATBX: antibiotic; NS: Normal Saline; a-FMT: Autologous-FMT; EK-FMT: Exercise combined with KGM intervention mice FMT; EXE-KGM: exercise combined with KGM intervention.

**Figure 14:**
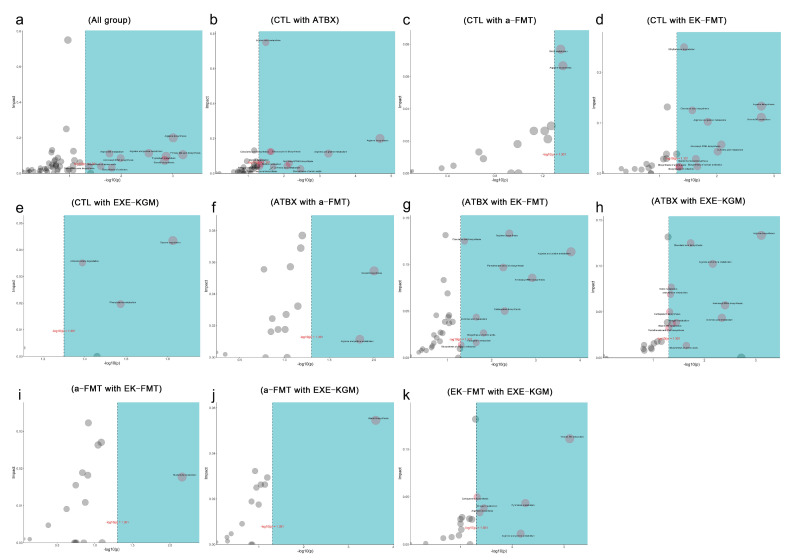
Topological analysis of different groups reveals the key biological pathways that play a significant role. (**a**) all group, (**b**) CTL with ATBX, (**c**) CTL with a-FMT, (**d**) CTL with EK-FMT, (**e**) CTL with EXE-KGM, (**f**) ATBX with a-FMT, (**g**) ATBX with EK-FMT, (**h**) ATBX with EXE-KGM, (**i**) a-FMT with EK-FMT, (**j**) a-FMT with EXE-KGM, (**k**) EK-FMT with EXE-KGM. Samples (*N* = 3) were colored by treatment. Pathways with biological significance screened by topological analysis are marked in red, with their names annotated following the vertical sequence of red circles. CTL: control; ATBX: antibiotic; NS: Normal Saline; a-FMT: Autologous-FMT; EK-FMT: Exercise combined with KGM intervention mice FMT; EXE-KGM: exercise combined with KGM intervention.

The ORA enrichment analysis indicates the metabolic pathways that significantly enrich the differences between groups of metabolites. Combined with the topological analysis diagram, it can show the importance of the metabolic pathways in biological processes. The metabolic pathway diagram simultaneously includes the metabolic pathways of metabolites and genes (KEGG complete metabolic pathways, without species distinction), and the translated proteins of gene expression usually play the role of enzymes in compound reactions, etc. Different colors correspond to different groups, indicating that in comparison with other groups, the metabolite content is higher in the corresponding group. After comparing the data of six groups, it was found that the enriched metabolic pathways included primary bile acid biosynthesis, arginine biosynthesis, tryptophan metabolism, steroid biosynthesis, arginine and proline metabolism, aminoacyl-tRNA biosynthesis, biosynthesis of cofactors, vitamin B6 metabolism, biosynthesis of amino acids, biosynthesis of unsaturated fatty acids, and steroid hormone biosynthesis (Figure 13a). The topological analysis shows (Figure 14a) that the top five metabolic pathways with higher impact values are Primary bile acid biosynthesis, arginine biosynthesis, tryptophan metabolism, arginine and proline metabolism, and vitamin B6 metabolism.

The results of pairwise comparisons among the groups showed that amino acid metabolism, particularly arginine biosynthesis and arginine and proline metabolism, as well as the metabolic pathways related to aminoacyl-tRNA biosynthesis, were the most enriched (Figure 13b–k and Figure 14b–k). Compared with the CTL group, the ATBX group showed the most significant changes. These pathways mainly included arginine biosynthesis, arginine and proline metabolism, and linoleic acid metabolism. Different intervention methods presented different metabolic pathway characteristics. The EK-FMT group significantly enriched the biosynthesis of various antibiotics and ethylbenzene degradation compared with the CTL group. In contrast, the EXE-KGM group enriched the styrene degradation, phenylalanine metabolism, and amino benzoate degradation pathways. When comparing the a-FMT group with the CTL group, its influence was relatively limited, and the enriched pathways included arginine biosynthesis and biotin metabolism, which also proved that autologous FMT had the least metabolic changes in mice. Comparisons between different intervention methods showed differences in their mechanisms of action. The differences between EK-FMT and a-FMT are mainly manifested in nucleotide metabolism. While the comparison between EK-FMT and EXE-KGM showed significant differences in vitamin B6 metabolism, pyrimidine metabolism, and carbapenem biosynthesis.

## 4. Discussion

In previous studies [17], the preventive and regulatory effects of exercise combined with KGM intervention on the prevention and correction of antibiotic-induced dysbiosis of the gut microbiome were first explored. The results showed that the exercise combined with the KGM intervention could effectively regulate dysbiosis of the gut microbiome. Therefore, in this study, the same KGM intervention concentration was adopted (2.5 g/L in drinking water). In terms of the microbiota structure alone, after two weeks and four weeks of antibiotic intervention, the Bray–Curtis distance between the ATBX group and the CTL group was the closest (Figure 7). This indicates that even without different intervention methods after stopping antibiotic intervention, the gut microbiome of mice still has a strong self-repair ability. Human experiments have shown that healthy adults have a spontaneous recovery phenomenon of gut microbiome after antibiotic intervention [50], and the recovery ability of the gut microbiome can protect the body from diseases related to microbiota imbalance [51]. However, some studies have shown that after antibiotic intervention, the effect of spontaneous recovery is not satisfactory [52]. This might be related to the type of antibiotics used, the age of the host, and the dietary structure [53,54].

To minimize physical stress on the mice while ensuring accurate volume and precise dosage, oral gavage was employed, as it enables more reliable results with milder stimulation and high reproducibility [55]. Oral gavage is the most commonly used route of administration in laboratory animal studies [56,57]. In a study evaluating intestinal permeability, the authors reported that gavage allows accurate quantitative delivery in vivo and represents a noninvasive, low-burden technique for assessing intestinal barrier function [58]. Although capsule-based administration can protect bacteria from gastric acid degradation, it may also lead to swallowing difficulties, particularly in mice, making this approach less applicable [59].

In this experiment, after two weeks of FMT intervention, although there was a significant difference in the Bray–Curtis distance between the a-FMT group and the CTL group, there was no significant difference in α diversity (Chao1, faith_pd, Shannon, Simpson) between the two groups. This confirmed that a-FMT still has a relatively positive effect and effectiveness on the restoration of the gut microbiome. In a randomized controlled clinical trial, autologous FMT significantly increased the recovery rate of gut microbiome after antibiotic treatment [60]. Another study also demonstrated that autologous FMT significantly accelerated the recovery of gut microbiome diversity and reduced the proliferation of potential pathogenic bacteria after antibiotic intervention [61].

Modifying the donor feces for FMT has become a new development direction for this method. Multi-donor FMT can induce clinical remission and endoscopic improvement in patients with active ulcerative colitis, and is associated with unique microbial changes in the results [62]. In a study examining the factors influencing exercise engagement in individuals with arthritis, the authors observed that 31% of arthritis patients were unwilling to participate in exercise, with pain identified as the predominant barrier to exercise within this population [63]. A separate investigation revealed that asthma patients may demonstrate diminished exercise tolerance, potentially attributable to exacerbation of asthma symptoms during physical activity or to deconditioning resulting from sedentary behavior, both of which may impede their participation in athletic activities or the preservation of physical health [64]. Importantly, asthmatic model rats that received fecal microbiota transplantation from healthy rats exhibited significant amelioration of asthma symptoms and marked restoration of intestinal short-chain fatty acid levels [65]. Based on previous research, exercise combined with KGM intervention can significantly regulate gut microbiome dysbiosis [17]. And at the end of the experiment, there was no significant difference in Bray–Curtis distance between the EK-FMT group and EXE-KGM group. This confirmed that the intervention effect on the gut microbiome can be preserved and transmitted. This result suggests that for those who did not engage in exercise after antibiotic intervention (such as patients who need bed rest due to fractures or other illnesses, or those with heart or lung diseases), even if these people cannot directly undergo exercise or other intervention methods, they can still receive similar intervention effects through FMT. Some studies have also reached similar conclusions, for example, young mice donor FMT improves the metabolic health of elderly mice and has higher α diversity [66]. One study pointed out that dietary intervention in the population after 6 months of intervention and collection of feces, the a-FMT treatment carried out during the recovery period may preserve the metabolic benefits induced by weight loss [67].

In this experiment, the animal experimental design did not incorporate a sedentary donor group without KGM supplementation as a control for the standard allogeneic FMT group. This experiment is an extension based on previous studies, focusing more on whether the combined effect of exercise and KGM can induce corresponding changes in the microbiota and whether such changes can be transmitted through FMT. The absence of this control may weaken the evidence supporting the beneficial effects of the EK-FMT group in specific populations. Accordingly, future investigations should optimize the experimental grouping design to guarantee the rigor and accuracy of research results.

After two weeks of nutrient and exercise intervention, *Akkermansia* significantly increased in the EXE-KGM group compared with the CTL group. However, unlike the predicted results, at the end of the experiment, the Bray–Curtis distance between the EXE-KGM group and the CTL group was significantly larger. This might be due to two reasons in this experiment: no exercise prevention intervention was carried out before the antibiotic intervention. and different levels of exercise intensity; therefore, compared with previous studies [17], the effect of the combined intervention was not particularly significant.

First, in previous studies [17], mice were subjected to two weeks of exercise combined with KGM intervention before antibiotic intervention. Although after the antibiotic intervention ended (D7), the combined intervention of exercise and KGM did not show significant protection for the gut microbiome. However, in combination with the experimental results of this study, we suppose that this kind of exercise prevention intervention has a two-sided effect that has been considered, and it may also increase the resilience of the intestinal microbiota to a certain extent. On the one hand, although exercise can reduce permeability, during antibiotic intervention, exercise may enhance the body’s absorption and utilization of antibiotics [68]. On the other hand, exercise can also alleviate the negative effects of antibiotics on the gut microbiome [69]. Moreover, due to the KGM intervention during the pre-treatment in previous studies, this might have led to the formation of a mucosal barrier in the intestine, reducing the impact of subsequent antibiotics on the gut microbiome [10]. These reasons resulted in the gut microbiome composition structure of the exercise combined with KGM intervention group being more similar to that of the control group at the end of the previous study. In fact, the initial state of the gut microbiome determines the recovery effect after antibiotic intervention [70]. A study pointed out that a high-fat and high-protein diet can exacerbate Clostridioides difficile infection in mice, while a high-carbohydrate diet can protect the intestinal health of mice [71]. Animal experiments have also shown that compared with a high-fat, low-fiber Western diet, mice on a regular diet experience a rapid and continuous recovery process after antibiotic intervention. This indicates that the different dietary patterns before antibiotic intervention have a significant impact on the adverse reactions caused by antibiotics. Therefore, this study suggested that a healthy lifestyle (good dietary patterns, long-term exercise habits, etc.) can play an important role in the recovery ability of the gut microbiome after antibiotic intervention. It is worth noting that C57BL/6J mice display limited generalizability in metabolic and immunological phenotypes and exhibit stable differences in metabolism, immune profiles, and gut microbiome relative to other substrains such as C57BL/6N [72]. The exclusive use of C57BL/6J may compromise the reproducibility and extrapolation of experimental outcomes. Furthermore, to avoid potential interference of estrogen on the experimental outcomes, only male mice were used in this study [72]. Overreliance on this single sub-strain further impedes the clinical translation of research conclusions to humans [73]. Therefore, the influence of strain selection, as well as sex-related factors, on the translational scope of research findings deserves more thorough examination. To this end, future studies are encouraged to integrate additional animal models, adopt multiple mouse strains, or perform preliminary human trials, thereby improving the translational value of the relevant findings.

Secondly, many studies have demonstrated the effectiveness of moderate intensity exercise [74,75,76]. Moderate-intensity exercise modulates exercise performance and intestinal function in mice, while enhancing beneficial microbial communities and epithelial barrier integrity and SCFAs production [77,78,79,80]. By contrast, high-intensity exercise is more inclined to induce marked gut dysbiosis and inflammatory responses [79,81]. Previous studies have demonstrated that natural KGM exerts a superior protective effect on the diversity and composition of fecal microbiota, thereby preventing microbial alterations triggered by overtraining [11]. In this experiment, to ensure that the mice continued to perform moderate-load exercise, endurance tests were conducted before and during the exercise intervention, so that the mice always performed moderate-intensity exercise. This is different from previous studies [17], in which, considering that antibiotics may affect the movement ability of mice [29], an inappropriate exercise intensity would lead to gut microbiome dysbiosis, reduced intestinal barrier function, and adverse effects on intestinal health [17]. We will determine the changes in exercise intensity and time based on the exercise performance of the mice during the exercise intervention. However, in this study, the mice were always kept to perform moderate exercise instead of the moderate-low exercise in our previous studies [17]. Therefore, the combined exercise intervention effect of this study did not significantly regulate gut microbiome dysbiosis. This may be attributed to the subtle alterations in gut microbial structure driven by changes in microbial diversity and the abundance of specific taxa following combined exercise intervention. Conventional microbiome analytical approaches may be insufficient to capture such subtle variations. Therefore, the application of machine learning models is required to achieve a more precise assessment of microbial diversity in further research [82,83]. This might suggest that during the recovery period of gut microbiome dysbiosis, engaging in moderate-low intensity or appropriately intense exercise is the most beneficial for regulating the gut microbiome [84]. Based on the above considerations, it is imperative to conduct further research to explore whether the comprehensive intervention of KGM combined with progressive loaded exercise can produce distinct effects in future studies.

In addition, after antibiotic intervention (D7), the Shannon index and Simpson index of the EK-FMT group showed no significant difference compared to the CTL group. The functional prediction analysis results showed that, although the Shannon and Simpson indices of the EK-FMT group on day 7 closely resembled those of the control group, according to the analysis of KEGG pathway level 2, the EK-FMT group on day 7 exhibited significant increases compared with the control group in several pathways, including “lipid metabolism”, “cell motility”, and “cellular community-prokaryotes”, etc. (Figure S7). This might be because, before the formal experiment, the feces of the mice in the a-FMT group needed to be collected in advance for autologous FMT. So before the formal experiment began, the six groups of mice had already been housed separately. Animals from the same cage exhibit comparable profiles due to shared environmental conditions. Due to the effect of cage [85], there were already slight differences in the gut microbiome of the mice during the adaptation period before the experiment began, resulting in inconsistent α diversity of the gut microbiome after antibiotic intervention.

Many studies have confirmed the regulatory effect of FMT on SCFAs [86] and its role in restoring the gut microbiome [87]. This study once again confirmed this point. Additionally, at D35, there was no significant difference in the concentration of various SCFAs between the ATBX group and the CTL group. However, the intervention of exercise combined with KGM caused very significant changes in the concentration of SCFAs in the feces of mice. Many studies have confirmed that exercise can increase the concentration of SCFAs in the feces of mice [80,88]. Even with excessive exercise, the intervention of exercise combined with KGM can still play a similar role [17]. This study also found that the intervention of exercise combined with KGM could significantly increase the concentration of SCFAs in the feces of mice, which is beneficial to the gut microbiome and the health of the host [89,90]. However, this study believes that the reason why no similar effect was produced on the D21 (two weeks of exercise combined with KGM intervention) might be that the gut microbiome dysbiosis caused by antibiotic intervention prevented the gut microbiome from functioning normally and thus was unable to provide energy metabolism substrates for exercise [91]. But after a certain period of recovery of the gut microbiome and adaptation to exercise, the ability of the gut microbiome to produce SCFAs was significantly improved. Notably, the intervention of exercise combined with FMT (EK-FMT group) in mice did not produce a similar effect, suggesting that the effect of exercise combined with KGM intervention in increasing SCFAs cannot be transmitted through FMT. As a study has shown [92], the gut microbiome of exercise donor mice could not fully replicate the similar exercise effects after FMT. This might require further confirmation by extending the duration of FMT. Furthermore, the changes brought about by exercise may depend on continuous physiological stimulation. Without continuous exercise stimulation, these changes may be temporary or even disappear [93].

Interestingly, in D49, except for the EXE-KGM group, the SCFAs in all other groups showed an upward trend compared to D35, even in the CTL group (Figure S2). A reasonable explanation is that over time, the mice gradually adapted to the feeding conditions, which might affect the production of SCFAs. Relevant studies have shown that the feeding environment can regulate SCFAs over time [94,95]. At the same time, no significant increase in SCFAs was found in the EXE-KGM group at D49, which is similar to the previously mentioned reason: exercise enhanced the elasticity of the mice’s gut microbiome, enabling it to adapt to the new feeding environment more quickly. After antibiotic intervention and recovery, it maintained the stability of SCFAs.

After conducting metagenomic sequencing analysis of the feces of the experimental endpoint mice, it was found that the a-FMT group enriched the amino acid synthesis pathway (KEGG: map00471 and map00473), which is consistent with the ability of the gut microbiome to precisely reconstruct the host nutritional metabolic network, as indicated in the literature [96]. Additionally, the a-FMT group successfully activated butyrate production (M00157), the pentose phosphate pathway (M00005), and the glycolysis module (M00049). This change is highly consistent with the enrichment of GH13 (α-amylase) and GH31 (α-glucosidase) carbohydrate-active enzymes in the CAZy database, and these changes may suggest that a-FMT has altered the energy metabolism characteristics of the mice. Moreover, the enzymes enriched in the CAZy database, which are present in these pathways, can participate in the degradation of dietary fibers and various carbohydrate structures, affecting the host’s health [97]. Furthermore, the CAZy database of the EK-FMT group showed enrichment of carbohydrate-active enzymes such as GH13, indicating that the combined intervention of donor exercise and KGM successfully transplanted the glucose metabolism-related functions into the mice.

This study also found that the EK-FMT group failed to fully replicate the metabolic advantages of the EXE-KGM group. For instance, in the KEGG modules, genes M00157 (butyrate production) and M00050 (gluconeogenesis) were absent, and the abundance of GH13 in the CAZy enzyme spectrum was significantly lower than that in the a-FMT and EK autologous groups. Additionally, the enrichment analysis by EggNOG revealed that the EK-FMT and a-FMT groups shared the enrichment of ENOG4107FTN (stress response protein), but the EK-FMT group lacked the specific GT113 (rhamnose glycosyltransferase) and other cell wall remodeling enzymes that were unique to the a-FMT group. This indicates that the colonization ability of the donor strain in the recipient’s intestine is limited, and there is a certain degree of rejection reaction [98]. In the second week of this experiment, the weight loss of mice in the EK-FMT group also proved the existence of the rejection reaction, and as the FMT process came to an end, the body weight of the mice in the EK-FMT group returned to normal. Moreover, although the results in Figure 10a show that the EK-FMT group only significantly enriched ARO_3000195, both this result and the relevant literature [99] suggest that FMT carries a certain risk of transferring antibiotic resistance genes. Overall, the metagenomic analysis indicated that the a-FMT group restored the intestinal metabolic balance by reconstructing the butyrate production module and the glycoside hydrolase network; the EXE-KGM group uniquely activated the D-aspartate metabolism and coenzyme regeneration pathways, while the allogeneic FMT (EK-FMT group) was limited by the rejection reaction and failed to fully transmit the intervention effects of combined exercise and KGM, and there is still a risk of horizontal transfer of drug resistance genes.

Although principal component analysis indicated that there were no significant differences in the overall composition of metabolites among the groups, and no differences were found in the top 20 metabolites and their pathway functions in terms of content among the groups, the random forest model, as well as single-dimensional statistical analysis, enrichment analysis, and other methods, revealed specific changes in some metabolites with significant differences. This may be explained by the fact that metagenomic and metabolomic analyses in this study were performed with three biological replicates per group because of cost constraints. This sampling condition imposes certain constraints on the interpretation of enrichment data and limits statistical resolution, which restricts the comprehensive identification of predictive factors related to microbial engraftment after FMT. For subsequent research, it is suggested to involve all samples for analysis to further enhance statistical robustness [100].

The ATBX group exhibited a severe disruption of the microbial-host co-metabolic balance. The content of 2,10-Dihydroxy-2,6,10-trimethyl-3,6,11-dodecatrien-5-one and CP-863187 in the ATBX group significantly decreased, and these two metabolites are closely related to the intestinal barrier and immune regulation [101]. This might indicate a certain degree of decline in the immune regulatory function of the ATBX group. Additionally, the ATBX group enriched for arginine biosynthesis precursors (C00062, C00064). Meanwhile, the CTL group maintained a balanced metabolic profile. This imbalance may result from the fact that the microbiota that originally participated in arginine degradation was cleared by antibiotics, leading to the accumulation of precursor metabolites in the host, thereby affecting the nitric oxide synthesis and immune regulation functions of the ATBX group mice [102].

Although the gut microbiome structure of the a-FMT group was the most similar to that of the CTL group, the EK-FMT and EXE-KGM groups could improve the metabolism of the gut microbiome by activating multiple metabolic pathways. Because the donors received the treatment of exercise combined with KGM intervention, the EK-FMT group showed a strong metabolic remodeling effect. The significant increase in valsaliclovir, H-isoleucine-tyrosine-hydroxy, and triptolide content was related to the enhanced host metabolic adaptability and anti-inflammatory effect [103]. EXE-KGM demonstrated a more comprehensive metabolic regulatory ability. It not only reduced the levels of pro-inflammatory metabolites such as 2-methyl-5-propylpyrazine but also enriched beneficial substances such as yin-Yanghuo C, indicating the formation of a synergistic anti-inflammatory mechanism [104]. Additionally, the EXE-KGM group significantly increased the level of prostaglandin E2. Prostaglandin E2 has been confirmed to be able to promote the regeneration of epithelial crypts and maintain the tight junction of the intestinal barrier under conditions such as intestinal injury caused by dextran sulfate sodium, chemotherapy, radiotherapy, colon surgery, or ischemia–reperfusion [105]. Moreover, the EK-FMT group significantly enriched the pathways related to antibiotic synthesis and exogenous substance degradation. This study suggests that the mice in the EK-FMT group actively inhibited the original intestinal microbiota by synthesizing various antibiotics and degrading potentially harmful substances, and occupied the ecological niche actively [106]. The EK-FMT group significantly activated the pathways related to antibiotic synthesis and exogenous substance degradation. This study hypothesizes that the mice in the EK-FMT group actively occupied ecological niches by synthesizing various antibiotics and decomposing potentially harmful substances, thereby reshaping the gut microbiome of the recipient mice and altering their metabolic characteristics.

The comprehensive analysis of metabolic pathways among the different groups revealed that arginine metabolism and vitamin B6 metabolism were the most fundamental and common metabolic modules. This finding is in line with current research on arginine metabolism and the gut microbiome. L-arginine metabolism occupies a central metabolic node at the host–microbe interface, acting not only as a substrate for both microbial and mammalian cells, but also shaping microbial energy use, colonization resistance, and immune signaling in the gut. Arginine is one of the 21 proteinogenic amino acids encoded in mammalian genomes [107]. Systemic extracellular arginine levels are maintained by the mitochondrial synthesis of citrulline in the small intestine from glutamate and glutamine [108]. Circulating citrulline is subsequently converted to arginine in peripheral tissues, primarily the kidneys [109]. During early development or infection, arginine synthesis alone is insufficient to meet metabolic demands [110]. Mitochondria play a central role in arginine metabolism, in addition to their well-established function in ATP production via oxidative phosphorylation [111]. Glutamate dehydrogenase catalyzes the synthesis of glutamate from the tricarboxylic acid cycle intermediate α-ketoglutarate (α-KG) and ammonium (NH_4_^+^). Carbamoyl phosphate synthase 1 (CPS1) carries out the ATP-dependent condensation of bicarbonate (HCO_3_^−^) and NH_4_^+^ to form carbamoyl phosphate (CP). To generate arginine, mitochondrial ornithine transcarbamylase (OTC) catalyzes the production of citrulline from CP and ornithine. Citrulline exported from mitochondria is then converted to arginine by cytosolic enzymes within the urea/citrulline cycle. Depending on cellular conditions and/or cell type, the resulting arginine can be utilized to support protein synthesis; to eliminate excess NH_4_^+^ through urea production; to serve as a precursor for the energy-buffering phosphagen creatine; to synthesize agmatine and polyamines; or to act as a signaling molecule regulating lysosomal amino acid release into the cytosol [112].

L-Arginine is the most nitrogen-rich amino acid, serving as a key precursor for the synthesis of nitrogen-containing metabolites and as an essential intermediate in the clearance of excess nitrogen. The guanidino group on the side chain of arginine possesses unique biochemical properties and plays a primary role in nitrogen excretion (urea), cellular signaling (nitric oxide), and energy buffering (phosphocreatine) [112]. In certain cell types, predominantly mature hepatocytes, macrophages, and non-proliferating T cells, arginine is taken up or produced in excess of cellular demands and is utilized to eliminate surplus nitrogen via the urea cycle [113,114,115]. To generate urea, cytosolic arginine is hydrolyzed back to ornithine by either cytosolic arginase 1 (ARG1) or mitochondrial arginase 2 (ARG2), with urea being released. Urea is subsequently transported out of the cell and excreted by the kidneys. Ornithine, in turn, is either re-imported into mitochondria to restart the cycle or used in the cytosol as a precursor for the biosynthesis of other products, such as polyamines [113]. Some cell types take up and/or synthesize arginine to support nitric oxide (NO) synthesis. In this cycle, arginine is converted to citrulline by nitric oxide synthase (NOS), generating NO. Citrulline can be recycled back to arginine through the activities of ASS1 and ASL, thereby completing the cycle [116]. The sole source of NO in mammalian cells is the catabolism of arginine by one of the members of the NOS family. NOS catalyzes the oxidation of arginine to NO and citrulline [117]. The functions of NO have been extensively described elsewhere. In brief, NO acts as a vasodilator in smooth muscle cells, functions as a retrograde neurotransmitter in synapses, and is secreted by myeloid cells to kill microbial pathogens [118].

Immune cells exhibit a strong dependence on arginine, and extracellular arginine is frequently depleted in inflammatory lesions, wounds, and tumors. This depletion results from increased arginine consumption by cells engaged in growth and repair, as well as from the secretion of arginase 1 (ARG1) by macrophages into the surrounding environment [119,120]. L-Arginine can be transported into cells by members of the solute carrier (SLC) transporter superfamily and G protein-coupled receptor 6A (GPCR6A). Once inside the cell, L-arginine is hydrolyzed to L-ornithine and urea by cytosolic ARG1, or transported into mitochondria, where it serves as a substrate for ARG2 [120].

The expression of ARG1 is regulated by both pro-inflammatory and anti-inflammatory cytokines. For example, T helper 2 (TH2) cell cytokines, such as IL-4 and IL-13, activate signal transducer and activator of transcription 6 (STAT6), which, together with CCAAT/enhancer-binding protein-β (C/EBPβ) or Krüppel-like factor 4 (KLF4), drives Arg1 expression in mice [121,122]. Furthermore, the anti-inflammatory cytokines IL-10 and transforming growth factor-β (TGF-β) also upregulate ARG1 expression by inducing a C/EBPβ isoform that directly binds to the Arg1 promoter [123,124]. Finally, the pro-inflammatory cytokines IL-6 and tumor necrosis factor (TNF) can upregulate Arg1 expression in a STAT3-dependent manner [125,126,127,128].

ARG2 is a mitochondrial enzyme expressed in various cell types in both mice and humans [123,129]. In mouse macrophages, ARG2 expression is induced by IL-10 during maturation and is negatively regulated by microRNA-155 (miR-155) [130,131]. In T cells, ARG2 is induced during the canonical activation program and is proposed to function as a negative-feedback regulator, preventing exacerbated immune responses through the depletion of L-arginine [132].

L-Arginine is essential for multiple cellular processes, including T cell activation, early B cell maturation, and osteoclast formation [133]. L-Arginine deficiency leads to cell cycle arrest and suppressed T cell proliferation. In the absence of L-arginine, T cells downregulate the CD3ζ chain of the T cell receptor (TCR), which may impair TCR signaling [133,134]. Conversely, arginine supplementation has been shown to promote development [135]. Dietary and microbial arginine can simultaneously influence intraluminal metabolism, immune responses, and microbial composition, underscoring its role as a pivotal mediator of host–microbiota crosstalk [136]. Consistent with this, recent work has shown that gut microbiome-derived arginine metabolism can mitigate intestinal injury through modulating microbial composition and host barrier function, and that fecal arginine profiles reflect the metabolic capacity of the resident microbiota in mouse models [137]. Furthermore, arginine administration has been demonstrated to remodel gut microbial communities in vivo, increasing the abundance of beneficial taxa such as Bifidobacterium and driving systemic immunometabolic effects, such as enhanced host defense via the gut-lung axis [138]. Moreover, early exposure of mice to antibiotics can alter the gut microbiome and metabolic characteristics, such as affecting amino acid metabolism [139]. Similar to arginine metabolism, vitamin B6 is one of the most fundamental molecules in the cells of living organisms. It is a key cofactor for various biochemical reactions that regulate basic cellular metabolism [140]. Some studies have also pointed out the close relationship between vitamin B6 and the gut microbiome. For example, vitamin B6 deficiency can damage the gut microbiome of rats as well as the host and microbial metabolites [141], and the gut microbiome can regulate the homeostasis of similar autistic behaviors in EphB6-deficient mice by mediating vitamin B6 [142]. The vitamin B6 metabolic disorder induced by microbiome dysbiosis is a new non-hypothalamic–pituitary–adrenal axis mechanism for chronic stress-related brain diseases, and supplementing vitamin B6 can alleviate weight loss, abnormal behavior, peripheral inflammation, and neuroinflammation in chronic stress rats [143]. The results of these studies and this research all indicate that arginine metabolism is closely related to the recovery of vitamin B6 and microbiome dysregulation.

## 5. Conclusions

In conclusion, this study suggests that pre-storage of fecal samples under healthy conditions may offer a potential strategy to address possible gut microbiome dysbiosis induced by various factors, not limited to antibiotics. The combination of exercise and KGM intervention can regulate gut microbiome dysbiosis by modulating metabolic pathways such as arginine metabolism and vitamin B6 metabolism. For those patients who suffer from serious diseases, refuse to exercise, or do not store their own healthy feces in advance, the effect can be achieved by transplanting the gut microbiome that has been modulated through a combination of exercise and KGM intervention. However, it is necessary to be cautious of the risk of rejection and the transfer of drug-resistant genes. In the future, it is necessary to conduct in-depth research on adjusting the exercise intensity after gut microbiome dysbiosis. Furthermore, the more effective application of FMT requires further validation in real-world clinical settings and adopts the most appropriate strategies for people with gut microbiome dysbiosis in different situations.

## Figures and Tables

**Table 1:** Exercise program for mice.

Group	Running Speed (m/min) and Running Time (min) of Mice During 1–3 Weeks of Exercise	Running Speed (m/min) and Running Time (min) of Mice During 4–6 Weeks of Exercise
EK-Donor	16 m/min, 30 min	17 m/min, 30 min
EXE-KGM	14 m/min, 30 min	14 m/min, 30 min

**Table 2:** Chromatographic gradient elution procedure.

Time (min)	A% (0.1% Formic Acid)	B% (Methanol)
0	98	2
1.5	98	2
3	15	85
10	0	100
10.1	98	2
11	98	2
12	98	2

## Data Availability

The data that support the findings of this study are openly available in the Mendeley Data database (https://data.mendeley.com/preview/cp6n94bbdx?a=76f0c42f-edb4-43c3-8c83-a354932be572 (accessed on 31 March 2026)). All other data generated or analyzed during this study are included in this published article and its Supplementary Information files. Additional data are available from the corresponding authors upon reasonable request.

## Supplementary Materials

The following supporting information can be downloaded at: https://www.mdpi.com/article/10.3390/nu18101544/s1, Table S1: The chemical compounds used in this work with information from NCBI PubChem compound database and the supplier; Table S2: The formula of AIN93 purified diet used in the study; Table S3: Histological scoring system; Table S4: The relative abundance in feces at different levels on day 7; Table S5: The relative abundance in feces at different levels on day 21; Table S6: The relative abundance in feces at different levels on day 35; Table S7: The relative abundance in feces at different levels on day 49; Figure S1: Body weight, dietary intake, and endurance profiles of the EK-Donor and EXE-KGM groups; Figure S2: The changes of different SCFAs in each group over time; Figure S3: Heatmap showing the correlation between gut microbiota at the phylum level and SCFA at different time points; Figure S4: Heatmap showing the correlation between gut microbiota at the family level and SCFA at different time points; Figure S5. Heatmap showing the correlation between gut microbiota at the genus level and SCFA at different time points; Figure S6: The analysis of non-targeted metabolites in feces of mice from each group; Figure S7: Stacked Bar Chart showing the effects of different intervention methods on the KEGG metabolic pathways in mice at day 7.
