# Supplementary material for: FMT from Exercise and Konjac Glucomannan Preconditioned Donors Rescues Antibiotic-Induced Dysbiosis with Enhanced Ecological Restoration in Mice
Source: Nutrients. 2026 May 13;18(10):1544. doi: 10.3390/nu18101544 (PMC13209768; doi:10.3390/nu18101544)
Supplement: Supplementary file 1 [file nutrients-18-01544-s001.zip › nutrients-4262060-supplementary.pdf]

Table S1. The chemical compounds used in this work with information from NCBI PubChem compound database and the supplier sources

Compound	CAS No.	CID	MW (g/mol)	InChIKey	IUPAC name	Source
Acetic acid	64-19-7	176	60.05	QTBSBXVTEAMEQO-UHFFFAOYSA-N	acetic acid	Aladdin® (Shanghai, China)
Propionic acid	79-09-4	1032	74.08	XBDQKXXYIPTUBI-UHFFFAOYSA-N	propanoic acid	Aladdin® (Shanghai, China)
n-butyric acid	107-92-6	264	88.11	FERIUCNNQQJTOY-UHFFFAOYSA-N	butynoic acid	Aladdin® (Shanghai, China)
iso-butyric acid	79-31-2	6590	88.11	KQNPFQTWMSNSAP-UHFFFAOYSA-N	2-methylpropanoic acid	Aladdin® (Shanghai, China)
n-valeric acid	109-52-4	7991	102.13	NQPDZGIKBAWPEJ-UHFFFAOYSA-N	pentanoic acid	Aladdin® (Shanghai, China)

Compound	CAS No.	CID	MW (g/mol)	InChIKey	IUPAC name	Source
iso-valeric acid	503-74-2	10430	102.13	GWYFCOCPABKNJV-UHFFFAOYSA-N	3-methylbutanoic acid	Aladdin® (Shanghai, China)
Ampicillin	7177-48-2	6249	349.4	*RXDALBZNGVATNY-CWLIKTDRSA-N*	(2S,5R,6R)-6-[[(2R)-2-amino-2-phenylacetyl]amino]-3,3-dimethyl-7-oxo-4-thia-1-azabicyclo[3.2.0]heptane-2-carboxylic acid	Macklin® (Shanghai, China)
Clindamycin hydrochloride	21462-39-5	16051951	461.4	AUODDLQVRAJAJM-XJQDNNTCSA-N	(2S,4R)-N-[(1S,2S)-2-chloro-1-[(2R,3R,4S,5R,6R)-3,4,5-trihydroxy-6-methylsulfanyloxan-2-yl]propyl]-1-methyl-4-propylpyrrolidine-2-carboxamide;hydrochloride	Macklin® (Shanghai, China)

Compound	CAS No.	CID	MW (g/mol)	InChIKey	IUPAC name	Source
streptomycin	57-92-1	19649	581.6	UCSJYZPVAKXKNQ-HZYVHMACSA-N	2-[(1R,2R,3S,4R,5R,6S)-3-(diaminomethylideneamino)-4-[(2R,3R,4R,5S)-3-[(2S,3S,4S,5R,6S)-4,5-dihydroxy-6-(hydroxymethyl)-3-(methylamino)oxan-2-yl]oxy-4-formyl-4-hydroxy-5-methyloxolan-2-yl]oxy-2,5,6-trihydroxycyclohexyl]	Macklin® (Shanghai, China)

	guanidine

Table S2. The formula of AIN93 purified diet used in the study

Ingredient	gram	kcal
Casein, 30Mesh	200	800
L-Cystine	3	12
Corn Starch	397	1590
Maltodextrin 10	132	528
Sucrose	100	400
Cellulose	50	0
Soybean Oil	70	630
t-Butylhydroquinone	0.014	0
Mineral Mix S10022M	35	0

Vitamin Mix V10037	10	40
Choline Bitartrate	2.5	0
Ingredient	gram	kcal
Total	1000	3850
Macronutrients		
Protein	20.0 (gram%)	20.3 (Kcal%)
Carbohydrate	64.0 (gram%)	63.9 (Kcal%)

Table S3. Histological scoring system

Histological score	Degree of inflammation	Infiltration of inflammatory	Degree of damage to the crypt	Crypt abscesses	Degree of submucosal edema	Reduction of goblet cells	Degree of epithelial hyperplasia
0	Normal	Normal	Normal	Normal	Normal	Normal	Normal
1	Mucosa	Unifocal	Basal 1/3 of crypt	Unifocal	Unifocal	Unifocal	Unifocal
2	Submucosa	Multifocal	Basal 2/3 of crypt	Multifocal	Multifocal	Multifocal	Multifocal

3	Muscular	Suffuse	Suffuse		Suffuse	Suffuse	Suffuse
4	Serous		Damage to the crypt and ulceration				

Table S4. The relative abundance in feces at different levels on day 7

D7	CTL	ATBX	NS	a-FMT	EK-FMT	EXE-KGM
**Phylum**						
Proteobacteria	8.77±9.28c	83.27±15.35abc	93.03±6.24a	93.22±9.85a	64.82±11.62bc	98.03±2.10a
Bacteroidota	59.94±10.90a	1.58±2.63b	0.75±0.48abc	1.31±2.54bc	9.40±3.10ab	0.23±0.16c
Firmicutes	27.74±7.38a	10.39±10.71abc	2.41±1.77bc	3.95±5.31abc	17.79±11.21ab	0.98±1.15c
Actinobacteriota	1.71±1.88	1.37±1.88	1.49±1.71	0.59±0.81	1.59±1.22	0.18±0.25
Verrucomicrobiota	1.09±2.14	1.40±3.39	0.63±0.63	0.41±0.95	0.48±1.17	0.18±0.25
unclassified	0.01±0.03b	0.80±1.02ab	0.14±0.14ab	0.32±0.42ab	2.69±1.57a	0.15±0.14ab
Planctomycetota	0.01±0.02	0.18±0.41	0.36±0.81	0.00±0.00	0.72±1.74	0.00±0.01
Campilobacterota	0.33±0.55a	0.00±0.00b	0.00±0.00b	0.00±0.00b	0.69±1.69ab	0.00±0.00b
Desulfobacterota	0.24±0.40a	0.46±1.07ab	0.00±0.00b	0.00±0.00b	0.00±0.00b	0.13±0.32ab

D7	CTL	ATBX	NS	a-FMT	EK-FMT	EXE-KGM
Halobacterota	0.00±0.00	0.15±0.17	0.10±0.18	0.06±0.14	0.53±0.48	0.00±0.00
Myxococcota	0.04±0.09	0.00±0.00	0.06±0.13	0.06±0.14	0.57±1.39	0.01±0.03
Nitrospinota	0.00±0.00	0.00±0.00	0.00±0.00	0.00±0.00	0.61±1.48	0.00±0.00
Patescibacteria	0.00±0.01	0.11±0.25	0.50±0.91	0.00±0.00	0.00±0.00	0.01±0.01
Fusobacteriota	0.00±0.00	0.00±0.00	0.40±0.90	0.00±0.00	0.00±0.00	0.00±0.00
Crenarchaeota	0.00±0.01	0.30±0.73	0.00±0.00	0.00±0.00	0.00±0.00	0.00±0.00
Acidobacteriota	0.00±0.01	0.00±0.00	0.07±0.15	0.06±0.11	0.00±0.00	0.07±0.18
Deinococcota	0.00±0.00	0.00±0.00	0.04±0.06	0.00±0.00	0.11±0.25	0.00±0.01
Deferribacterota	0.07±0.09a	0.00±0.00b	0.01±0.02ab	0.00±0.00b	0.00±0.00b	0.00±0.00b
Cyanobacteria	0.02±0.03a	0.00±0.00b	0.00±0.00ab	0.00±0.01b	0.00±0.00b	0.00±0.00b
Synergistota	0.00±0.00	0.00±0.00	0.00±0.00	0.00±0.00	0.00±0.00	0.01±0.02
Other	0.02±0.04	0.00±0.00	0.00±0.00	0.00±0.01	0.00±0.00	0.00±0.00
**Family**						
Burkholderiaceae	0.23±0.22c	40.19±32.30abc	83.67±14.17ab	79.11±24.91ab	6.00±3.53bc	95.21±3.79a

D7	CTL	ATBX	NS	a-FMT	EK-FMT	EXE-KGM
Pseudomonadaceae	0.08±0.09c	27.58±11.57ab	5.09±3.35abc	10.23±12.15abc	38.76±13.11a	1.92±1.58c
Muribaculaceae	50.24±15.79a	1.06±2.03ab	0.09±0.15b	0.65±1.60b	5.80±5.52ab	0.17±0.17b
Lachnospiraceae	7.69±3.73a	1.08±1.69ab	0.39±0.36ab	1.10±1.37ab	3.49±3.94ab	0.18±0.22b
Sphingomonadaceae	0.12±0.22c	3.74±2.06ab	0.86±0.70abc	1.31±1.38abc	7.51±1.65a	0.27±0.20bc
Erysipelotrichaceae	9.77±7.33a	0.08±0.11ab	0.00±0.00b	0.28±0.48b	0.34±0.79ab	0.14±0.28ab
Enterobacteriaceae	4.80±4.93a	1.06±1.06ab	0.14±0.23ab	0.56±0.76ab	2.95±4.34ab	0.08±0.10b
Bacillaceae	0.00±0.01	0.57±0.77	0.15±0.32	0.32±0.72	5.37±9.65	0.10±0.20
Streptococcaceae	0.03±0.04ab	5.47±10.30a	0.60±0.89ab	0.01±0.02b	0.33±0.81ab	0.05±0.10ab
Lactobacillaceae	2.84±1.37	0.29±0.52	0.48±0.67	0.63±1.39	0.85±1.54	0.11±0.13
Erysipelatoclostridiaceae	4.67±2.91a	0.00±0.00b	0.00±0.00b	0.03±0.07b	0.00±0.00b	0.00±0.00b

D7	CTL	ATBX	NS	a-FMT	EK-FMT	EXE-KGM
Moraxellaceae	0.00±0.00b	1.55±2.09a	0.40±0.60ab	0.39±0.45ab	2.11±2.97a	0.02±0.01ab
unclassified	0.06±0.07b	0.80±1.02ab	0.16±0.18ab	0.40±0.46ab	2.72±1.56a	0.17±0.13ab
Tannerellaceae	3.83±1.71a	0.23±0.56b	0.00±0.00b	0.00±0.00b	0.00±0.00b	0.00±0.00b
Xanthomonadaceae	0.03±0.03	3.92±9.16	0.08±0.12	0.00±0.00	0.00±0.00	0.00±0.00
Akkermansiaceae	1.07±2.09	1.40±3.39	0.51±0.63	0.41±0.95	0.48±1.17	0.18±0.25
Beijerinckiaceae	0.00±0.00b	2.00±3.19a	0.15±0.13ab	0.23±0.33ab	1.06±1.54a	0.13±0.19ab
Rikenellaceae	2.47±5.19a	0.00±0.00b	0.00±0.00b	0.00±0.00b	1.02±2.45ab	0.00±0.00b
Sutterellaceae	3.06±3.31a	0.00±0.00b	0.00±0.00b	0.00±0.01b	0.00±0.00b	0.00±0.01b
Prevotellaceae	1.50±1.22	0.25±0.60	0.25±0.35	0.49±1.01	0.17±0.40	0.00±0.01
Other	7.49±2.22ab	8.71±6.68ab	6.97±8.09ab	3.83±4.16ab	21.03±14.45a	1.25±1.51b
**Genus**						
Burkholderia_Caballeronia_Paraburkholderia	0.15±0.23c	38.70±33.24abc	82.90±15.23ab	78.22±24.58abc	1.08±1.15bc	95.04±3.89a

D7	CTL	ATBX	NS	a-FMT	EK-FMT	EXE-KGM
Pseudomonas	0.08±0.09c	27.31±11.56ab	5.08±3.35abc	10.23±12.15abc	38.76±13.11a	1.92±1.58bc
Muribaculaceae	48.79±16.68a	1.05±2.03ab	0.09±0.16b	0.65±1.60b	5.46±5.75ab	0.16±0.16b
unclassified	5.20±5.07ab	3.71±2.36ab	1.14±1.19b	1.59±1.71ab	5.57±2.60a	0.43±0.27b
Sphingomonas	0.02±0.03b	3.74±2.06ab	0.86±0.70b	1.31±1.38b	7.47±1.65a	0.27±0.20b
Ralstonia	0.06±0.03b	1.49±1.01ab	0.44±0.51ab	0.45±0.29ab	3.97±3.07a	0.17±0.19b
Bacillus	0.00±0.01	0.57±0.77	0.15±0.32	0.31±0.72	5.37±9.65	0.10±0.20
Streptococcus	0.03±0.04ab	5.47±10.30a	0.60±0.89ab	0.01±0.02b	0.33±0.81ab	0.05±0.10ab
Lachnoclostridium	5.62±2.81a	0.00±0.00b	0.01±0.03b	0.00±0.00b	0.00±0.00b	0.01±0.01ab
Lactobacillus	2.84±1.37	0.29±0.52	0.48±0.67	0.63±1.39	0.85±1.54	0.11±0.13
Erysipelatoclostridium	4.67±2.91a	0.00±0.00b	0.00±0.00b	0.00±0.00b	0.00±0.00b	0.00±0.00b
Dubosiella	4.48±3.42a	0.02±0.04ab	0.00±0.00b	0.00±0.00b	0.00±0.00b	0.12±0.25ab

Ileibacterium	4.29±5.23a	0.05±0.10ab	0.00±0.00b	0.00±0.00b	0.00±0.00b	0.00±0.01b
Acinetobacter	0.00±0.00b	1.45±1.87a	0.34±0.51b	0.38±0.44b	2.07±2.98a	0.01±0.02b
Parabacteroides	3.82±1.71a	0.23±0.56b	0.00±0.00b	0.00±0.00b	0.00±0.00b	0.00±0.00b
Akkermansia	1.07±2.09	1.40±3.39	0.51±0.63	0.41±0.95	0.48±1.17	0.18±0.25
Xanthomonas	0.00±0.00	3.86±9.02	0.08±0.12	0.00±0.00	0.00±0.00	0.00±0.00
Methylobacterium_Methylorubrum	0.00±0.00b	1.87±3.25a	0.15±0.13ab	0.23±0.33ab	1.06±1.54a	0.13±0.19ab
Parasutterella	3.06±3.31a	0.00±0.00b	0.00±0.00b	0.00±0.01b	0.00±0.00b	0.00±0.01b
Escherichia_Shigella	0.00±0.00	0.40±0.95	0.11±0.24	0.01±0.02	2.50±4.13	0.03±0.05
Other	15.79±10.06a	8.37±5.96ab	7.04±8.09ab	5.55±5.24ab	25.03±15.64a	1.26±1.52b

Data are shown in average ± standard deviation. Different letters indicate the significant difference among different groups for the same index, ANOVA with LSD or Tamhane T2 post-hoc test. N = 6 or 5. CTL: control; ATBX: antibiotic; NS: Normal Saline; a-FMT: Autologous-FMT; EK-FMT: Exercise combined with KGM intervention mice FMT; EXE-KGM: exercise combined with KGM intervention.

Table S5. The relative abundance in feces at different levels on day 21

D21	CTL	ATBX	NS	a-FMT	EK-FMT	EXE-KGM
**Phylum**						
Bacteroidota	63.23±10.89a	57.45±9.43ab	60.18±9.82ab	42.88±10.57b	35.20±8.39b	17.12±9.06c
Firmicutes	28.66±8.07	29.13±10.90	32.01±9.63	31.79±14.29	25.50±7.42	24.61±17.12
Verrucomicrobiota	3.38±3.51c	8.94±5.73c	2.36±2.34c	20.59±16.10bc	35.99±8.95ab	55.88±15.95a
Proteobacteria	1.19±0.68b	3.29±1.45a	4.07±1.52a	0.55±0.33b	1.41±0.69b	1.39±0.39b
Actinobacteriota	1.76±0.94	0.78±0.39	1.29±0.68	0.79±0.65	0.57±0.41	0.94±0.76
Desulfobacterota	1.18±1.17a	0.37±0.48ab	0.02±0.03b	0.45±0.71ab	1.12±0.95a	0.03±0.06b
Campilobacterota	0.24±0.31ab	0.03±0.09b	0.06±0.04ab	2.48±4.72a	0.46±1.10ab	0.00±0.00ab
Cyanobacteria	0.14±0.12ab	0.00±0.00b	0.00±0.00b	0.15±0.12a	0.19±0.41ab	0.04±0.05ab
Deferribacterota	0.19±0.27a	0.00±0.00b	0.00±0.00b	0.18±0.21a	0.00±0.00b	0.00±0.00b
Patescibacteria	0.02±0.03ab	0.00±0.00b	0.00±0.00b	0.12±0.10a	0.11±0.13ab	0.00±0.00b
unclassified	0.01±0.01	0.00±0.00	0.01±0.01	0.01±0.02	0.00±0.01	0.00±0.00
Fusobacteriota	0.00±0.00b	0.00±0.00b	0.00±0.00b	0.00±0.00b	0.00±0.00b	0.02±0.03a
Halobacterota	0.00±0.01	0.00±0.00	0.00±0.00	0.00±0.00	0.00±0.00	0.00±0.00
**Family**						

D21	CTL	ATBX	NS	a-FMT	EK-FMT	EXE-KGM
Muribaculaceae	59.75±10.11a	52.20±9.66ab	54.79±8.24a	36.21±8.30b	33.14±7.71b	15.12±7.91c
Akkermansiaceae	3.38±3.51c	8.94±5.73c	2.36±2.34c	20.59±16.10bc	35.99±8.95ab	55.88±15.95a
Lachnospiraceae	8.44±5.67	11.02±8.08	7.98±4.69	8.45±5.75	10.49±7.64	4.59±6.31
Erysipelotrichaceae	6.39±3.53	4.52±1.64	14.32±10.79	3.09±1.48	8.39±5.95	15.09±16.97
Lactobacillaceae	9.73±7.28ab	10.84±6.54a	7.73±1.75ab	14.16±14.80ab	3.85±2.16ab	2.08±1.10b
Bacteroidaceae	1.86±1.09ab	4.45±2.47a	2.75±1.81ab	1.96±1.72ab	1.01±1.07b	1.64±1.06ab
Sutterellaceae	1.05±0.68ab	2.80±1.59a	2.98±1.27a	0.32±0.20b	0.74±0.39ab	1.09±0.28ab
Erysipelatoclostridiaceae	0.81±1.71	1.84±1.22	1.24±1.10	1.54±0.59	1.54±0.34	0.96±0.67
Eggerthellaceae	0.96±0.43	0.57±0.33	0.91±0.72	0.72±0.61	0.31±0.22	0.93±0.76
Rikenellaceae	0.15±0.11ab	0.00±0.00a	0.17±0.18ab	3.83±3.41a	0.06±0.07ab	0.00±0.01a
Oscillospiraceae	1.31±0.88a	0.18±0.16ab	0.25±0.29ab	1.84±1.66a	0.10±0.06b	0.50±0.71ab
Ruminococcaceae	0.58±0.33ac	0.15±0.09bc	0.03±0.03b	2.08±1.93a	0.42±0.17abc	0.56±0.34abc
Desulfovibrionaceae	1.18±1.17a	0.37±0.48abc	0.02±0.03bc	0.45±0.71abc	1.12±0.95ac	0.00±0.01b
Prevotellaceae	0.70±1.19ab	0.00±0.00b	1.63±1.82a	0.77±0.77a	0.26±0.14ab	0.00±0.00b
Tannerellaceae	0.24±0.15	0.79±0.53	0.82±0.73	0.12±0.15	0.73±0.58	0.35±0.32
Helicobacteraceae	0.24±0.31ab	0.03±0.09b	0.06±0.04ab	2.48±4.72a	0.01±0.01b	0.00±0.00b

D21	CTL	ATBX	NS	a-FMT	EK-FMT	EXE-KGM
Clostridia_UCG_014	0.53±0.51a	0.07±0.07ab	0.14±0.07ab	0.07±0.15b	0.11±0.13ab	0.58±0.59a
Enterobacteriaceae	0.04±0.07b	0.17±0.10ab	0.89±0.96a	0.07±0.14b	0.24±0.14ab	0.20±0.19ab
Atopobiaceae	0.60±0.47a	0.13±0.08abc	0.16±0.04abc	0.06±0.04c	0.25±0.19ac	0.00±0.01b
unclassified	0.06±0.03ab	0.06±0.06b	0.18±0.18ab	0.08±0.08ab	0.42±0.24a	0.09±0.07ab
Other	2.00±0.47a	0.86±0.47ab	0.58±0.27b	1.11±0.71ab	0.83±0.45ab	0.32±0.22b
**Genus**						
Muribaculaceae	56.53±9.42a	50.45±9.28ab	54.26±8.10a	35.07±8.01b	30.20±7.70bc	15.06±7.87c
Akkermansia	3.38±3.51b	8.94±5.73b	2.36±2.34b	20.59±16.10b	35.99±8.95ab	55.88±15.95a
Lactobacillus	9.73±7.28ab	10.84±6.54a	7.73±1.75ab	14.16±14.80ab	3.85±2.16ab	2.08±1.10b
Dubosiella	3.87±2.84	2.66±1.63	13.00±10.19	2.41±1.20	4.64±4.44	15.07±16.98
Lachnoclostridium	0.12±0.10b	9.93±7.83a	6.89±4.34a	0.82±0.86ab	10.19±7.67a	4.19±6.30ab
Bacteroides	1.86±1.09ab	4.45±2.47a	2.75±1.81ab	1.96±1.72ab	1.01±1.07b	1.64±1.06ab
unclassified	3.01±1.90a	0.54±0.26ab	1.25±1.04ab	4.59±3.67a	0.78±0.36ab	0.55±0.83b
Muribaculum	3.21±1.56a	1.75±0.55ac	0.54±0.18bc	1.14±0.45abc	2.93±3.22ac	0.06±0.06b
Lachnospiraceae_NK4A136_group	5.06±3.05a	0.03±0.06bc	0.08±0.10abc	3.48±3.20ab	0.02±0.03c	0.02±0.04bc
Parasutterella	1.05±0.68ab	2.80±1.59a	2.98±1.27a	0.32±0.20b	0.74±0.39ab	1.09±0.28ab

D21	CTL	ATBX	NS	a-FMT	EK-FMT	EXE-KGM
Erysipelatoclostridium	0.80±1.72	1.73±1.22	1.22±1.09	1.54±0.59	1.52±0.34	0.92±0.65
Ileibacterium	1.68±1.63ac	1.71±1.47a	0.41±0.50abc	0.06±0.06bc	0.76±0.69ac	0.00±0.01b
Faecalibaculum	0.65±0.44ab	0.00±0.00b	0.65±0.47ab	0.37±0.33ab	2.93±1.59a	0.00±0.00b
Enterorhabdus	0.82±0.39	0.51±0.29	0.74±0.73	0.61±0.48	0.25±0.18	0.89±0.73
Parabacteroides	0.24±0.15	0.79±0.53	0.82±0.73	0.12±0.15	0.73±0.58	0.35±0.32
Helicobacter	0.24±0.31ab	0.03±0.09b	0.06±0.04ab	2.48±4.72a	0.01±0.01b	0.00±0.00b
Desulfovibrio	1.18±1.17a	0.22±0.30ab	0.00±0.00b	0.38±0.67ab	1.00±0.90a	0.00±0.01b
Rikenellaceae_RC9_gut_group	0.00±0.00b	0.00±0.00b	0.15±0.19ab	2.11±1.95a	0.04±0.06ab	0.00±0.00b
Alistipes	0.15±0.11ab	0.00±0.00b	0.02±0.02ab	1.72±1.55a	0.02±0.02ab	0.00±0.01b
Prevotellaceae_UCG_001	0.70±1.19a	0.00±0.00b	0.37±0.42ab	0.77±0.77a	0.12±0.08ab	0.00±0.00b
Other	5.73±1.56a	2.60±0.93b	3.73±2.11ab	5.31±3.84ab	2.29±0.79b	2.19±0.88b

Data are shown in average ± standard deviation. Different letters indicate the significant difference among different groups for the same index, ANOVA with LSD or Tamhane T2 post-hoc test. N = 6 or 5. CTL: control; ATBX: antibiotic; NS: Normal Saline; a-FMT: Autologous-FMT; EK-FMT: Exercise combined with KGM intervention mice FMT; EXE-KGM: exercise combined with KGM intervention.

Table S6. The relative abundance in feces at different levels on day 35

D35	CTL	ATBX	NS	a-FMT	EK-FMT	EXE-KGM
**Phylum**						
Bacteroidota	74.93±11.35	73.93±7.50	84.17±10.33	84.17±7.75	84.76±5.15	81.00±11.37
Firmicutes	23.73±11.12	21.34±5.42	13.72±9.80	13.59±7.69	14.10±5.19	16.70±10.43
Desulfobacterota	0.63±0.61ab	4.21±4.46a	0.94±0.81ab	1.63±1.77a	0.02±0.03b	1.72±2.51ab
Actinobacteriota	0.42±0.28	0.36±0.18	0.19±0.12	0.24±0.23	0.11±0.11	0.23±0.33
Proteobacteria	0.23±0.20	0.44±0.84	0.31±0.28	0.12±0.09	0.06±0.08	0.30±0.22
Patescibacteria	0.07±0.08	0.11±0.28	0.35±0.28	0.10±0.12	0.27±0.23	0.34±0.83
Campilobacterota	0.02±0.04	0.03±0.03	0.07±0.06	0.72±1.29	0.52±0.52	0.02±0.05
Verrucomicrobiota	0.06±0.16	0.08±0.15	0.24±0.24	0.06±0.07	0.12±0.10	0.08±0.10
Cyanobacteria	0.00±0.00b	0.03±0.07ab	0.01±0.01ab	0.01±0.01ab	0.07±0.10a	0.00±0.00b
Deferribacterota	0.01±0.01	0.06±0.10	0.04±0.09	0.02±0.03	0.00±0.00	0.00±0.00
unclassified	0.01±0.01	0.01±0.02	0.00±0.00	0.00±0.01	0.00±0.00	0.00±0.01
**Family**						
Muribaculaceae	71.71±10.61	71.47±6.28	82.11±10.35	79.62±5.95	83.01±4.80	78.44±11.17
Lachnospiraceae	14.10±13.70	12.57±4.69	8.19±11.02	7.81±7.85	4.64±3.69	8.51±7.99

D35	CTL	ATBX	NS	a-FMT	EK-FMT	EXE-KGM
Erysipelotrichaceae	3.37±2.38	2.28±2.87	1.68±1.90	1.26±1.04	6.07±6.58	4.40±9.24
Lactobacillaceae	3.98±2.85	4.56±4.67	1.26±0.77	2.20±2.34	1.75±1.63	1.82±1.54
Desulfovibrionaceae	0.63±0.61ab	4.21±4.46a	0.94±0.81ab	1.63±1.77a	0.02±0.03b	1.72±2.51ab
Oscillospiraceae	1.36±1.35	1.12±0.60	1.34±2.16	1.24±1.05	1.00±1.13	0.92±1.07
Prevotellaceae	0.45±0.35	0.77±0.71	0.86±0.52	2.33±2.36	0.64±0.46	1.41±0.56
Bacteroidaceae	0.81±0.38	0.97±0.84	0.70±0.67	0.95±0.48	0.26±0.22	0.50±0.33
Rikenellaceae	0.53±0.33	0.58±0.31	0.15±0.21	0.58±0.38	0.31±0.20	0.13±0.13
Tannerellaceae	0.03±0.03b	0.11±0.11ab	0.35±0.13ab	0.69±0.51a	0.55±0.37a	0.52±0.35a
Marinifilaceae	1.39±0.80a	0.02±0.05b	0.00±0.00b	0.00±0.00b	0.00±0.00b	0.00±0.00b
*Clostridia*_UCG_014	0.24±0.22	0.23±0.21	0.07±0.04	0.48±0.39	0.14±0.06	0.23±0.35
Ruminococcaceae	0.11±0.06	0.37±0.18	0.12±0.13	0.18±0.13	0.09±0.10	0.31±0.37
Eggerthellaceae	0.32±0.18	0.33±0.18	0.06±0.04	0.19±0.23	0.07±0.06	0.14±0.17
Staphylococcaceae	0.07±0.07	0.03±0.01	0.90±1.03	0.13±0.24	0.03±0.02	0.05±0.06
Sutterellaceae	0.23±0.20	0.08±0.07	0.31±0.28	0.11±0.08	0.06±0.07	0.28±0.21
Saccharimonadaceae	0.02±0.02ab	0.00±0.00b	0.35±0.28a	0.10±0.12ab	0.27±0.23a	0.00±0.00b
Helicobacteraceae	0.02±0.04ab	0.01±0.02b	0.06±0.07ab	0.06±0.11ab	0.52±0.52a	0.00±0.01b

D35	CTL	ATBX	NS	a-FMT	EK-FMT	EXE-KGM
Acholeplasmataceae	0.08±0.11ab	0.01±0.01ab	0.00±0.00b	0.00±0.00b	0.07±0.06a	0.31±0.70ab
Akkermansiaceae	0.00±0.00b	0.00±0.00b	0.24±0.24a	0.06±0.07ab	0.08±0.10ab	0.04±0.06ab
Other	0.55±0.26	0.28±0.15	0.32±0.07	0.38±0.16	0.44±0.34	0.25±0.23
**Genus**						
Muribaculaceae	67.39±9.31	68.12±6.66	75.03±10.29	69.69±7.95	67.10±6.49	65.25±13.96
Muribaculum	4.32±2.61bc	3.36±0.88c	7.08±0.85abc	9.93±3.08abc	15.90±2.48a	13.19±5.72ab
Lachnospiraceae_NK4A136_group	8.44±7.67	6.36±2.05	5.24±7.24	3.34±3.96	2.32±1.97	4.55±4.56
unclassified	4.98±5.03	5.01±1.92	2.81±3.28	4.01±3.85	2.04±1.62	2.84±2.88
Lactobacillus	3.98±2.85	4.56±4.67	1.26±0.77	2.20±2.34	1.75±1.63	1.82±1.54
Ileibacterium	1.53±1.23	1.63±2.01	1.29±1.44	0.28±0.32	3.42±3.67	2.44±5.21
Desulfovibrio	0.62±0.61ab	4.21±4.46a	0.94±0.81ab	1.63±1.77a	0.00±0.01b	1.71±2.51ab
Alloprevotella	0.13±0.17b	0.61±0.59ab	0.73±0.43ab	1.55±2.09ab	0.34±0.30ab	0.91±0.51a
Bacteroides	0.81±0.38	0.97±0.84	0.70±0.67	0.95±0.48	0.26±0.22	0.50±0.33
Allobaculum	0.74±0.64ab	0.00±0.00c	0.00±0.01bc	0.00±0.00c	2.27±2.51a	0.27±0.62abc
Dubosiella	0.54±0.33	0.55±0.80	0.29±0.37	0.43±0.39	0.18±0.21	0.82±1.85
Roseburia	0.52±0.73ab	0.88±0.60a	0.26±0.45ab	0.14±0.09ab	0.03±0.04b	0.76±1.08ab

D35	CTL	ATBX	NS	a-FMT	EK-FMT	EXE-KGM
Parabacteroides	0.03±0.03b	0.11±0.11ab	0.35±0.13ab	0.69±0.51a	0.55±0.37a	0.52±0.35a
Prevotellaceae_UCG_001	0.32±0.39	0.16±0.13	0.13±0.13	0.78±0.73	0.29±0.19	0.50±0.42
Lachnospiraceae_FCS020_group	0.28±0.29	0.54±0.41	0.23±0.29	0.30±0.30	0.07±0.03	0.44±0.64
Faecalibaculum	0.49±0.33	0.04±0.05	0.06±0.08	0.19±0.19	0.14±0.19	0.83±1.57
Lachnoclostridium	0.26±0.20	0.21±0.14	0.13±0.16	0.35±0.32	0.36±0.39	0.20±0.18
Odoribacter	1.39±0.80a	0.02±0.05b	0.00±0.00b	0.00±0.00b	0.00±0.00b	0.00±0.00b
Clostridia_UCG_014	0.24±0.22	0.23±0.21	0.07±0.04	0.48±0.39	0.14±0.06	0.23±0.35
Alistipes	0.53±0.33	0.00±0.01	0.11±0.20	0.53±0.34	0.13±0.09	0.03±0.01
Other	2.45±0.83	2.44±1.03	3.31±0.78	2.53±0.85	2.69±1.23	2.19±1.21

Data are shown in average ± standard deviation. Different letters indicate the significant difference among different groups for the same index, ANOVA with LSD or Tamhane T2 post-hoc test. N = 6 or 5. CTL: control; ATBX: antibiotic; NS: Normal Saline; a-FMT: Autologous-FMT; EK-FMT: Exercise combined with KGM intervention mice FMT; EXE-KGM: exercise combined with KGM intervention.

Table S7. The relative abundance in feces at different levels on day 49

D49	CTL	ATBX	NS	a-FMT	EK-FMT	EXE-KGM
**Phylum**						
Bacteroidota	52.58±12.92	33.84±20.29	44.20±27.51	46.10±19.61	56.05±21.84	48.46±16.80
Firmicutes	41.37±15.30	38.38±18.23	52.07±26.21	48.91±19.77	33.62±22.53	47.10±16.15
Verrucomicrobiota	0.27±0.52b	22.99±28.62a	0.02±0.01b	0.03±0.06b	0.01±0.02b	0.28±0.59ab
Desulfobacterota	3.54±2.84	0.78±1.30	0.89±1.73	3.08±3.16	5.12±3.25	1.62±1.92
Actinobacteriota	1.60±0.50	2.30±2.05	0.74±0.41	0.70±0.34	1.13±0.63	0.91±0.36
Patescibacteria	0.14±0.10ab	0.53±0.21ab	0.93±0.86ab	0.09±0.08b	2.04±1.69a	0.46±0.78ab
Campilobacterota	0.04±0.03	0.06±0.06	0.09±0.15	0.75±1.27	1.24±1.60	0.66±0.67
Proteobacteria	0.31±0.07	0.27±0.16	0.68±0.41	0.23±0.15	0.47±0.30	0.47±0.42
Deferribacterota	0.05±0.05ab	0.54±1.00a	0.19±0.42ab	0.09±0.14ab	0.02±0.03ab	0.00±0.00b
Cyanobacteria	0.07±0.08ab	0.30±0.29a	0.16±0.10ab	0.02±0.03b	0.29±0.31a	0.03±0.03ab
unclassified	0.00±0.01	0.01±0.02	0.03±0.07	0.00±0.00	0.01±0.03	0.00±0.01
Acidobacteriota	0.01±0.02	0.00±0.00	0.00±0.00	0.00±0.00	0.00±0.01	0.00±0.00
Planctomycetota	0.00±0.01	0.00±0.00	0.00±0.00	0.00±0.00	0.00±0.00	0.00±0.00
Myxococcota	0.00±0.00	0.00±0.00	0.00±0.01	0.00±0.00	0.00±0.00	0.00±0.00

D49	CTL	ATBX	NS	a-FMT	EK-FMT	EXE-KGM
Hydrogenedentes	0.00±0.01	0.00±0.00	0.00±0.00	0.00±0.00	0.00±0.00	0.00±0.00
Fusobacteriota	0.00±0.00	0.00±0.00	0.00±0.00	0.00±0.00	0.00±0.00	0.00±0.00
Dependentiae	0.00±0.00	0.00±0.00	0.00±0.00	0.00±0.00	0.00±0.00	0.00±0.00
Elusimicrobiota	0.00±0.00	0.00±0.00	0.00±0.00	0.00±0.00	0.00±0.00	0.00±0.00
Chloroflexi	0.00±0.00	0.00±0.00	0.00±0.00	0.00±0.00	0.00±0.00	0.00±0.00
Deinococcota	0.00±0.00	0.00±0.00	0.00±0.00	0.00±0.00	0.00±0.00	0.00±0.00
**Family**						
Muribaculaceae	49.12±12.05	31.20±18.72	39.75±23.72	43.22±18.22	54.77±21.12	44.12±14.69
Lactobacillaceae	16.65±11.97	11.30±10.75	36.68±29.00	34.49±18.88	14.15±22.10	23.76±15.36
Lachnospiraceae	12.41±5.86ab	14.17±12.13ab	8.00±5.94ab	3.61±2.07b	10.75±1.46a	11.63±8.85ab
Erysipelotrichaceae	6.87±7.71	5.33±3.26	1.13±0.70	7.27±8.35	4.48±2.25	4.67±5.78
Akkermansiaceae	0.26±0.52ab	22.99±28.62a	0.02±0.01b	0.28±0.37ab	0.46±0.65ab	0.41±0.60ab
Desulfovibrionaceae	3.54±2.84	0.78±1.30	0.89±1.73	3.08±3.16	5.12±3.25	1.62±1.92
*Clostridia*_UCG_014	1.21±0.74	2.91±2.15	3.09±3.08	0.96±0.92	0.89±0.56	1.78±1.18
Oscillospiraceae	2.24±2.21	1.41±1.38	0.87±0.83	1.33±1.12	1.14±1.02	2.05±0.87
Eggerthellaceae	1.38±0.47	1.77±1.64	0.71±0.39	0.64±0.30	0.84±0.52	0.77±0.39

D49	CTL	ATBX	NS	a-FMT	EK-FMT	EXE-KGM
Ruminococcaceae	0.77±0.57	1.49±1.14	0.35±0.39	0.50±0.54	0.86±0.27	1.63±1.12
Prevotellaceae	0.87±0.47	0.33±0.35	1.37±1.00	1.25±1.36	0.29±0.40	1.63±0.94
Bacteroidaceae	0.90±0.45	0.99±1.19	1.67±2.24	0.76±0.53	0.45±0.42	0.78±0.68
Saccharimonadaceae	0.14±0.10ab	0.53±0.21ab	0.93±0.86ab	0.09±0.08b	2.04±1.69a	0.46±0.78ab
Rikenellaceae	0.99±0.69	0.58±0.40	0.77±0.59	0.53±0.34	0.37±0.26	0.75±0.73
Helicobacteraceae	0.04±0.03	0.06±0.06	0.09±0.15	0.75±1.27	1.24±1.60	0.66±0.67
RF39	0.63±0.53	0.45±0.25	0.61±0.73	0.11±0.11	0.55±0.51	0.54±0.33
Tannerellaceae	0.29±0.22	0.21±0.20	0.58±0.47	0.33±0.27	0.16±0.16	1.18±2.37
Sutterellaceae	0.13±0.08	0.13±0.11	0.39±0.48	0.11±0.08	0.21±0.36	0.26±0.41
Atopobiaceae	0.16±0.09ab	0.46±0.40a	0.01±0.02b	0.06±0.04ab	0.25±0.18a	0.10±0.12ab
[Eubacterium]_coprostanoligenes_group	0.00±0.00b	0.48±0.44a	0.30±0.30ab	0.07±0.06ab	0.10±0.17ab	0.08±0.15ab
Other	1.38±0.67	2.43±1.56	1.80±0.89	0.81±0.69	1.33±0.62	1.24±0.76
**Genus**						
Muribaculaceae	45.42±11.95b	25.12±18.95ab	36.21±22.17b	39.40±17.62b	43.60±18.66a	37.23±14.23ab
Lactobacillus	16.65±11.97	11.30±10.75	36.68±29.00	34.49±18.88	14.15±22.10	23.76±15.36
Muribaculum	3.70±0.82	6.07±2.45	3.53±2.02	3.82±0.80	11.15±2.91	6.88±3.51

D49	CTL	ATBX	NS	a-FMT	EK-FMT	EXE-KGM
Lachnospiraceae_NK4A136_group	6.59±3.02ab	6.92±6.28ab	4.16±2.91ab	1.81±1.22b	7.51±1.17a	7.01±7.00ab
unclassified	5.66±3.46	8.06±6.31	2.87±1.65	2.43±1.73	3.21±1.40	4.97±2.47
Akkermansia	0.26±0.52ab	22.99±28.62a	0.02±0.01b	0.72±1.27ab	2.71±4.11ab	1.19±2.16ab
Desulfovibrio	3.53±2.84ab	0.76±1.28ab	0.85±1.74b	3.07±3.14a	5.10±3.25ab	1.58±1.94ab
Dubosiella	1.49±1.39	2.52±1.75	0.48±0.43	5.60±6.35	0.97±0.37	1.09±1.02
Clostridia_UCG_014	1.21±0.74	2.91±2.15	3.09±3.08	0.96±0.92	0.89±0.56	1.78±1.18
Ileibacterium	1.69±2.92	2.12±1.68	0.53±0.50	1.16±1.71	2.66±1.91	2.18±3.73
Enterorhabdus	1.28±0.44	1.66±1.55	0.63±0.34	0.55±0.25	0.70±0.43	0.70±0.36
Bacteroides	0.90±0.45	0.99±1.19	1.67±2.24	0.76±0.53	0.45±0.42	0.78±0.68
Candidatus_Saccharimonas	0.14±0.10ab	0.53±0.21ab	0.93±0.86ab	0.09±0.08b	2.04±1.69a	0.46±0.78ab
Alloprevotella	0.43±0.13ab	0.46±0.45ab	0.72±0.57ab	0.95±1.15ab	0.17±0.20b	1.14±0.58a
Allobaculum	3.20±5.08a	0.00±0.00ab	0.12±0.15ab	0.13±0.27ab	0.17±0.15ab	0.00±0.00b
Incertae_Sedis	0.57±0.44	0.39±0.27	0.11±0.09	0.36±0.43	0.57±0.28	0.97±0.80
Helicobacter	0.04±0.03	0.08±0.05	0.11±0.14	0.75±1.27	1.24±1.60	0.66±0.67
RF39	0.63±0.53	0.45±0.25	0.61±0.73	0.11±0.11	0.55±0.51	0.54±0.33
Parabacteroides	0.29±0.22	0.21±0.20	0.58±0.47	0.33±0.27	0.16±0.16	1.18±2.37

D49	CTL	ATBX	NS	a-FMT	EK-FMT	EXE-KGM
Alistipes	0.77±0.47	0.18±0.14	0.32±0.28	0.42±0.29	0.25±0.20	0.50±0.28
Other	5.53±3.26	6.49±3.34	6.06±3.84	2.89±1.23	4.43±1.81	6.79±4.61

Data are shown in average ± standard deviation. Different letters indicate the significant difference among different groups for the same index, ANOVA with LSD or Tamhane T2 post-hoc test. N = 6 or 5. CTL: control; ATBX: antibiotic; NS: Normal Saline; a-FMT: Autologous-FMT; EK-FMT: Exercise combined with KGM intervention mice FMT; EXE-KGM: exercise combined with KGM intervention.

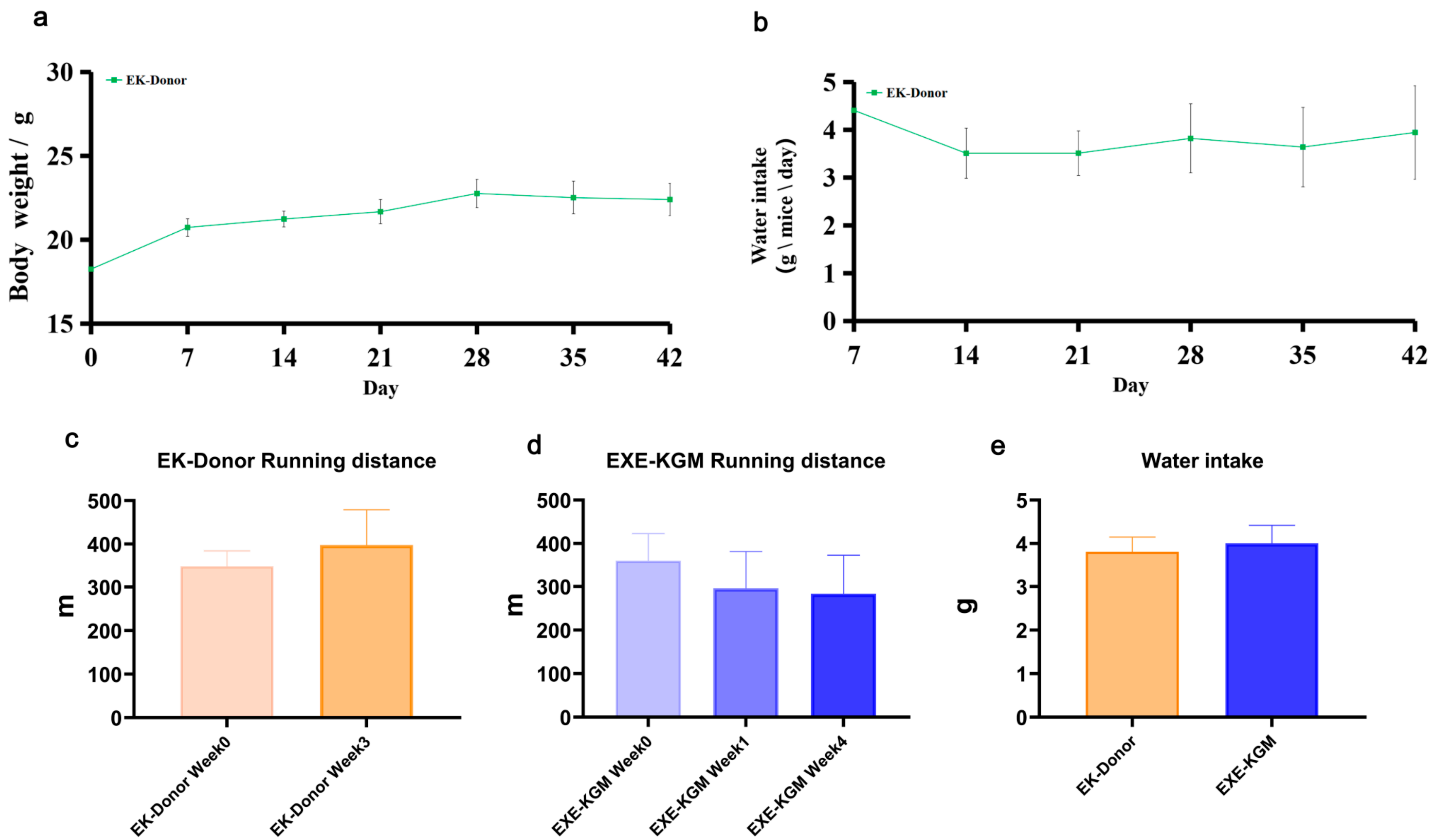
a
EK-Donor
Body weight / g
30
25
20
15
0
7
14
21
28
35
42
Day
b
EK-Donor
Water intake
(g \ mice \ day)
5
4
3
2
1
0
7
14
21
28
35
42
Day
c
EK-Donor Running distance
m
500
400
300
200
100
0
EK-Donor Week0
EK-Donor Week3
d
EXE-KGM Running distance
m
500
400
300
200
100
0
EXE-KGM Week0
EXE-KGM Week1
EXE-KGM Week4
e
Water intake
g
5
4
3
2
1
0
EK-Donor
EXE-KGM

Figure S1. (a) the body weight of EK-Donor group, (b) the average daily water intake of mice of EK-Donor group, (c) the endurance comparison of mice in the EK-Donor group, (d) the endurance comparison of mice in the EXE-KGM group, (e) the average daily water intake of mice in the EK group and the EXE-KGM group. ANOVA with Bonferroni or Tamhane T2 post-hoc test or Kruskal-Wallis test. Average of eight or six samples, error bars represent standard deviation at N = 8 or 6. EK-Donor: Exercise+KGM-Donor, EXE-KGM: exercise combined with KGM intervention.

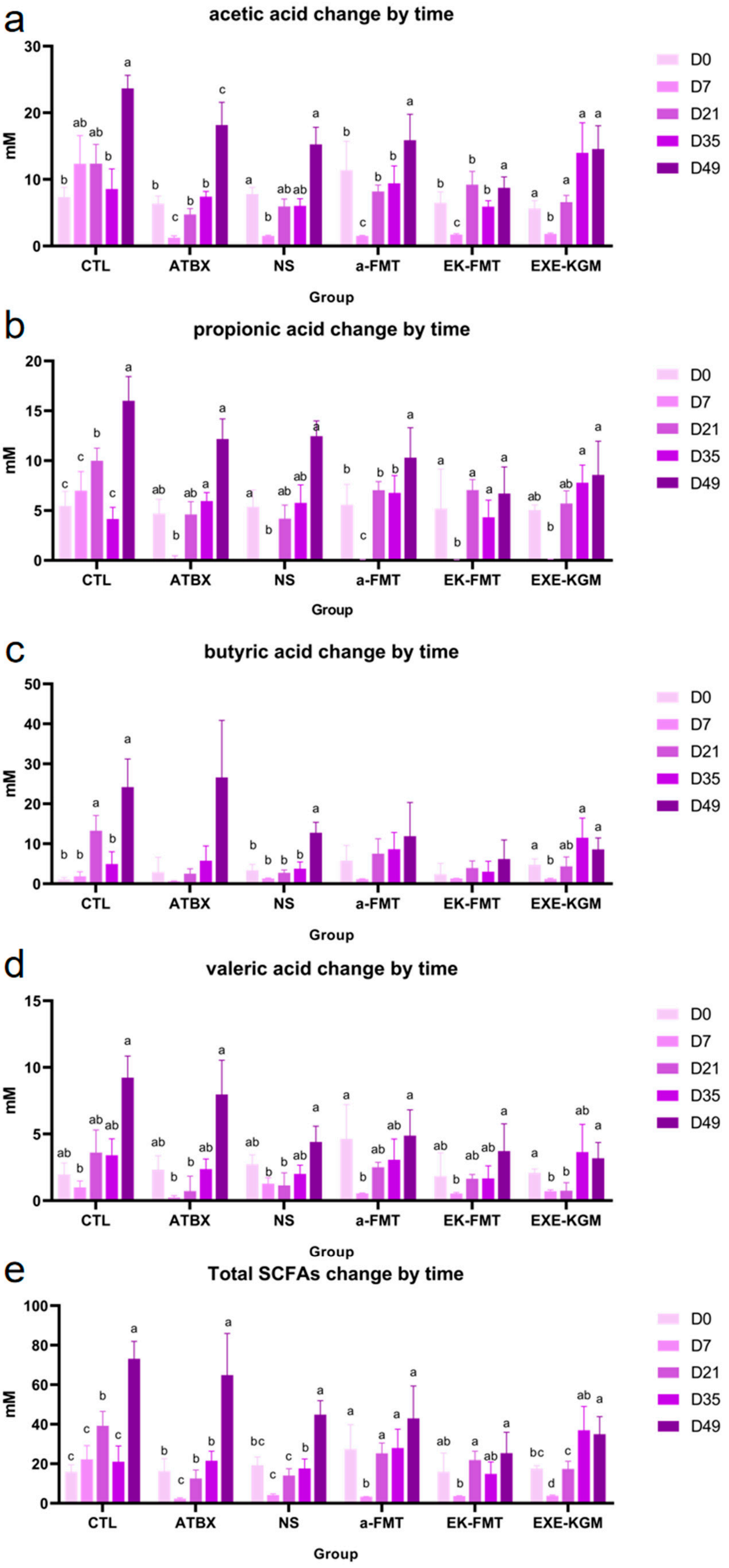

Figure S2. The changes of different SCFAs in each group over time (a) acetic acid; (b)

propionic acid; (c) butyric acid; (d) valeric acid; (d) total SCFAs. Different letters indicate the significant difference among different groups for the same index, ANOVA with Bonferroni or Tamhane T2 post-hoc test or Kruskal-Wallis test. Average of six or five samples, error bars represent standard deviation at N = 6 or 5. CTL: control; ATBX: antibiotic; NS: Normal Saline; a-FMT: Autologous-FMT; EK-FMT: Exercise combined with KGM intervention mice FMT; EXE-KGM: exercise combined with KGM intervention.

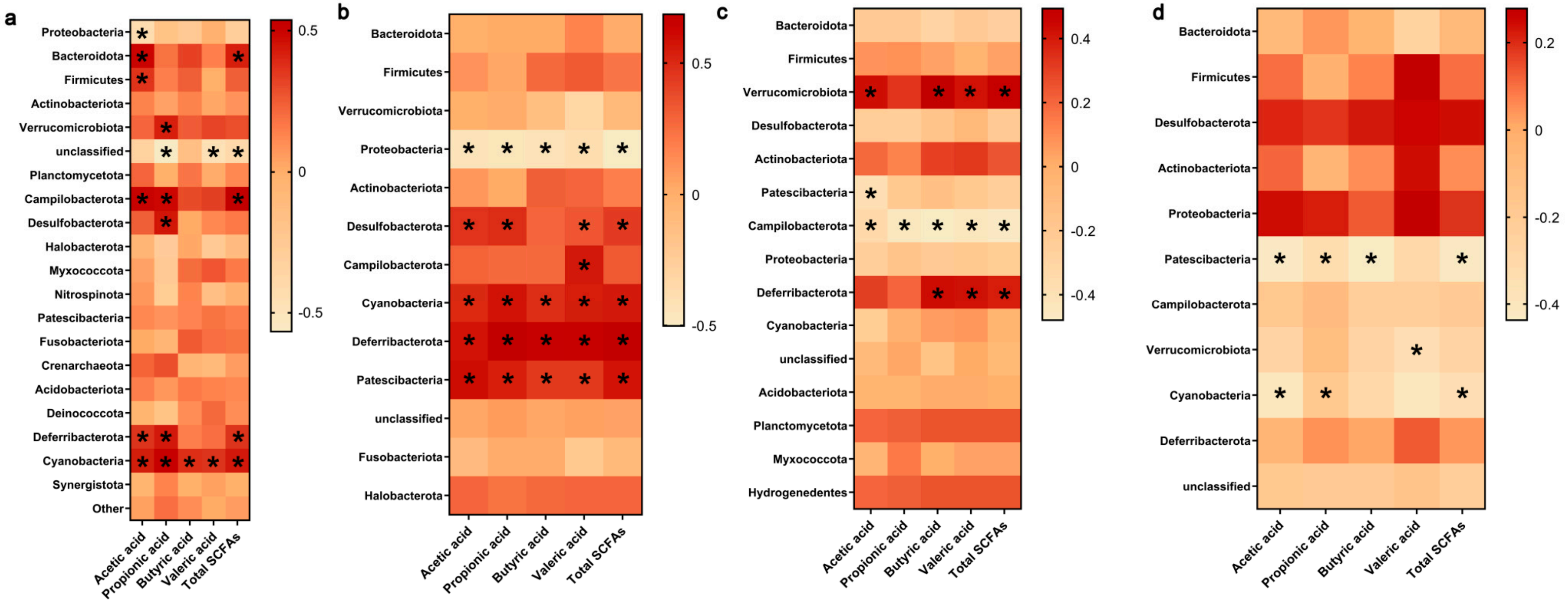

Figure S3. Heatmap showing the correlation between gut microbiota at the phylum level and SCFA at different time points (a) D7; (b) D21; (c) D35; (d) D49. Spearman correlation analysis, * $p < 0.05$.

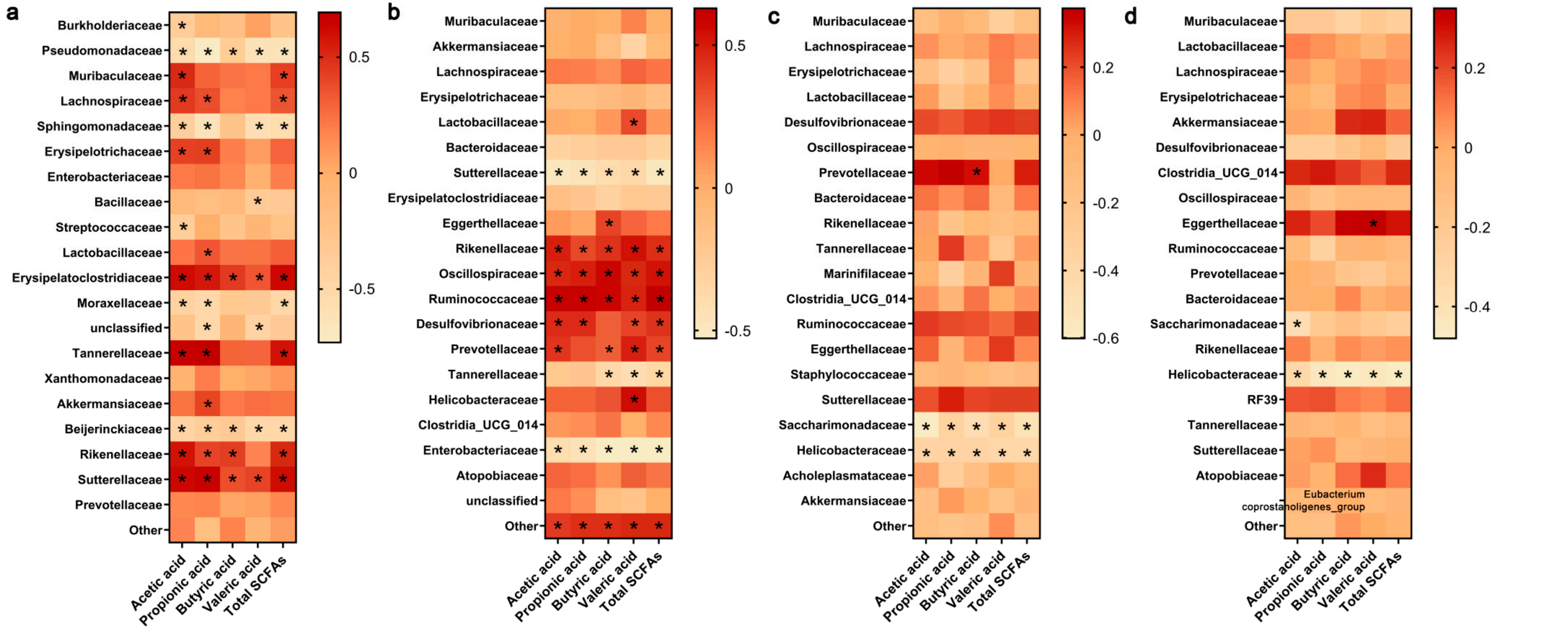

Figure S4. Heatmap showing the correlation between gut microbiota at the family level and SCFA at different time points (a) D7; (b) D21; (c) D35; (d) D49. Spearman correlation analysis, * $p < 0.05$.

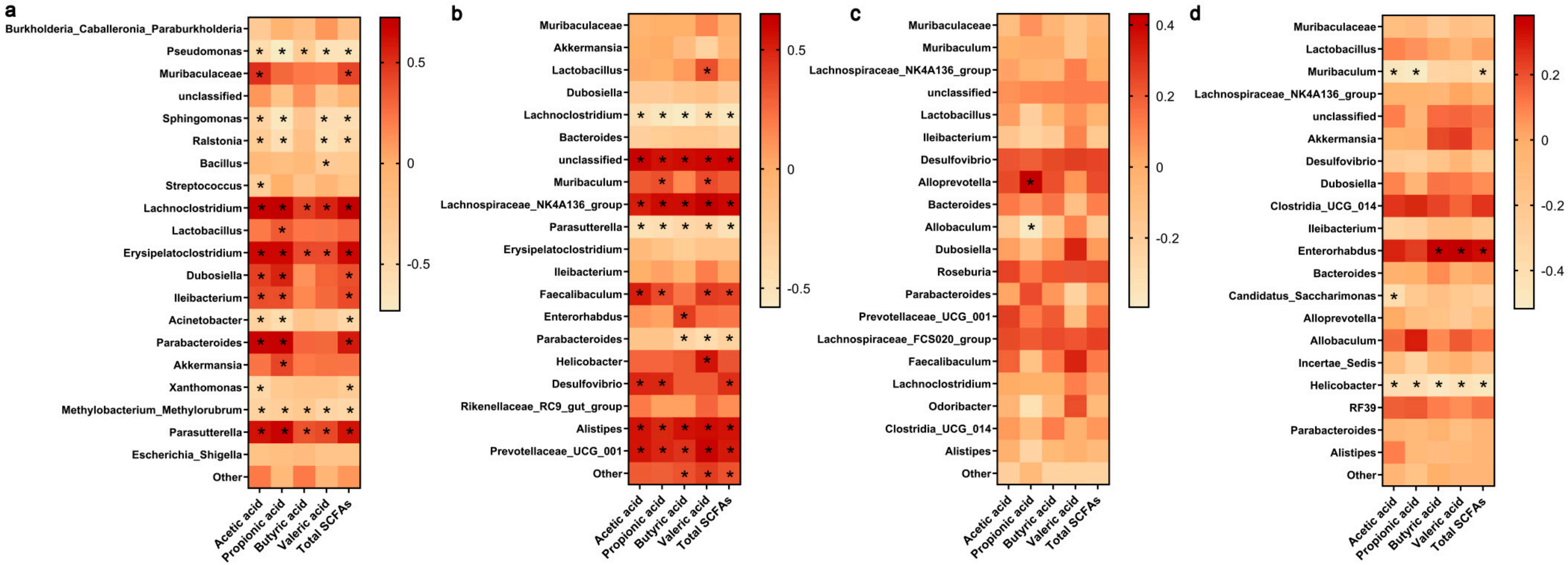

Figure S5. Heatmap showing the correlation between gut microbiota at the genus level and SCFA at different time points (a) D7; (b) D21; (c) D35; (d) D49. Spearman correlation analysis, * $p < 0.05$.

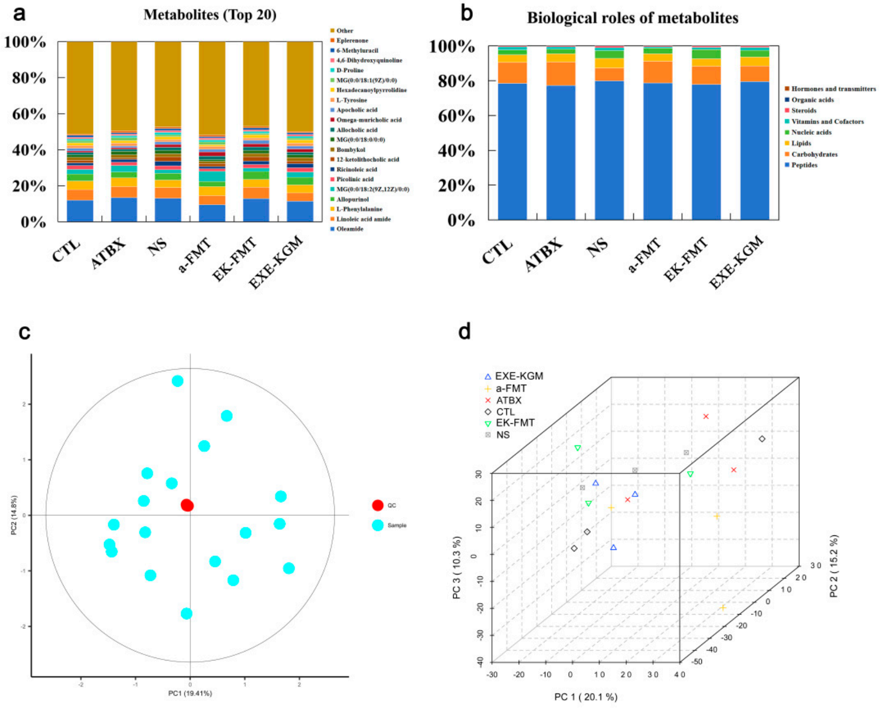

Figure S6: The analysis of non-targeted metabolites in feces of mice from each group. (a) Bar chart showing the percentage accumulation of the top 20 metabolites, (b) Bar chart showing the percentage accumulation of metabolites playing biological roles, (c) Quality control sample PCA plot, (d), PCA-3D graph of structural differences in metabolite composition. Samples (N = 3) were colored by treatment. CTL: control; ATBX: antibiotic; NS: Normal Saline; a-FMT: Autologous-FMT; EK-FMT: Exercise combined with KGM intervention mice FMT; EXE-KGM: exercise combined with KGM intervention.

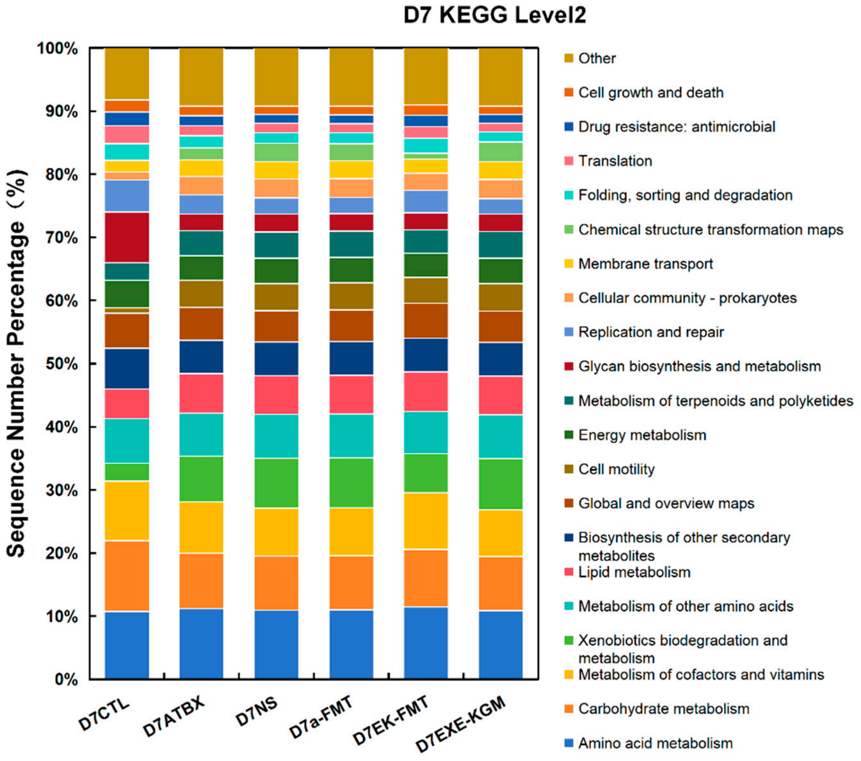

Figure S7. Stacked Bar Chart showing the effects of different intervention methods on the KEGG metabolic pathways in mice at day 7. Samples (N = 3) were colored by treatment. CTL: control; ATBX: antibiotic; NS: Normal Saline; a-FMT: Autologous-FMT; EK-FMT: Exercise combined with KGM intervention mice FMT; EXE-KGM: exercise combined with KGM intervention.
